# Transcriptome-wide characterization of the endogenous miR-34A-p53 tumor suppressor network

**DOI:** 10.18632/oncotarget.10417

**Published:** 2016-07-06

**Authors:** Nardin Samuel, Gavin Wilson, Badr Id Said, Anna Pan, Genevieve Deblois, Nicholas W. Fischer, Roumiana Alexandrova, Guillermo Casallo, Tara Paton, Mathieu Lupien, Jean Gariepy, Daniele Merico, Thomas J. Hudson, David Malkin

**Affiliations:** ^1^ Department of Medical Biophysics, Faculty of Medicine, University of Toronto, Toronto, Canada; ^2^ Genetics and Genome Biology Program, The Hospital for Sick Children, Toronto, Canada; ^3^ Ontario Institute for Cancer Research, Toronto, Canada; ^4^ Department of Molecular Genetics, Faculty of Medicine, University of Toronto, Toronto, Canada; ^5^ Princess Margaret Cancer Centre, University Health Network, Toronto, Canada; ^6^ Department of Physical Sciences, Sunnybrook Research Institute, Toronto, Canada; ^7^ The Centre for Applied Genomics (TCAG), Program in Genetics and Genome Biology, The Hospital for Sick Children, Toronto, Canada; ^8^ Division of Hematology/Oncology, Department of Pediatrics, The Hospital for Sick Children, University of Toronto, Toronto, Canada

**Keywords:** miR-34A, p53, TP53, cell cycle, non-coding RNA

## Abstract

microRNA-34A is a critical component of the p53 network and expression of miR- 34A is down-regulated by promoter hypermethylation or focal deletions in numerous human cancers. Although miR-34A deregulation may be an important driver in cancer, the endogenous role of this microRNA in cellular homeostasis is not well characterized. To address this knowledge gap, we aimed to determine the transcriptional landscape of the miR-34A-p53 axis in non-transformed cells. Using primary skin-derived fibroblast cell lines from patients who developed childhood cancers, and who harbor either germline *TP53* mutations or are *TP53* wild type, we sought to characterize the transcriptional response to miR-34A modulation. Through transcriptome-wide RNA-Sequencing, we show for the first time that in human non- transformed cells harboring *TP53* mutations, miR-34A functions in a noncanonical manner to influence noncoding RNA networks, including RNA components of the minor (U12) spliceosome, as well as *TP53*-dependent and independent epigenetic pathways. miR- 34A-regulated transcripts include known cell cycle mediators and abrogation of miR-34A leads to a *TP53*-dependent increase in the fraction of cells in G2/M. Collectively, these results provide a framework for understanding the endogenous role of the miR-34A signaling axis and identify novel transcripts and pathways regulated by the essential miR-34A-p53 tumor suppressor network.

## INTRODUCTION

miR-34A is the first identified microRNA (miRNA) found to be involved in the p53 regulatory network [1, 2]. p53-dependent regulation of *miR-34A* is mediated by a canonical p53 binding site that occurs within 30 kb of the *miR-34A* transcription start site at the 1p36 locus [3]. Studies have shown that miR-34A can induce variable effects on p53 transcriptional activity, either positively by targeting p53 inhibitor transcripts such as MDM4, SIRT1, MTA2, HDAC1 and YY1, or negatively by directly targeting TP53 mRNA [4]. Although the net effect of miR- 34A over-expression on p53 levels is highly cell context dependent, studies have provided evidence of an essential positive feedback loop between p53 and miR- 34A in mediating tumor suppression [5].

Akin to p53, miR-34A deregulation is pervasive in human cancer. miR-34A inactivation by focal loss of 1p36 or promoter hypermethylation has been reported in a multitude of human malignancies [2, 6, 7] (Table S1). Moreover, miR-34A has been shown to be repressed in cancer stem cell populations [8]. Owing to its established role in cancer, synthetic miR-34A mimics are currently in Phase I clinical trials for hepatocellular carcinoma, renal cell carcinoma, melanoma, lung cancers, and a number of hematologic malignancies (NCT01829971). Although it is well known that miR-34A deregulation may be an important driver in cancer, the exact mechanisms of its role in cellular homeostasis have remained elusive [9]. Previous studies have aimed to characterize the cellular effects of miR-34A in cancer cell lines and identified candidate effectors of the miR-34A transcriptional network [10]. However, there is a lack of studies on the transcriptional pathways that govern endogenous miR- 34A function in non-transformed cells. Clarification of the mechanisms by which endogenous miR-34A functions as a tumor suppressor, and the vulnerabilities to tumorigenesis that occur as a result of its deregulation, are therefore needed.

To address this gap, we aimed to characterize the transcriptional landscape of the miR-34A-p53 axis in human primary non-transformed cells. Patients harboring germline mutations in *TP53*, consistent with Li-Fraumeni Syndrome (LFS), are highly susceptible to developing cancer, particularly in childhood and young adulthood [11]. Primary skin-derived fibroblast cell lines from LFS patients provide the opportunity to study miR-34A modulation in the context of *TP53* mutation. Cell lines from patients who developed pediatric malignancies, but were *TP53* wild-type were utilized for comparison.

By exploring the transcriptional response to miR- 34A modulation in *TP53* mutant and *TP53* wild- type cell lines, we report the first global profile of the miR- 34A-dependent transcriptome in human non-transformed cells and demonstrate that miR-34A differentially regulates transcripts in the background of mutant and wild-type p53. We further characterize the impact of these gene expression changes on cell viability and cell cycle. These analyses reveal that miR-34A is a central node in numerous p53-dependent and independent networks, including previously unreported regulation of replication-dependent histone genes, long intergenic non-coding RNAs (lincRNAs) and components of the U12-dependent spliceosome. These results provide a framework for understanding the basal function of miR- 34A and demonstrate that miR-34A is essential to the maintenance of cellular homeostasis.

## RESULTS

### Establishing the transcriptional profile of non-transformed mutant p53 cells

**Figure 1:**
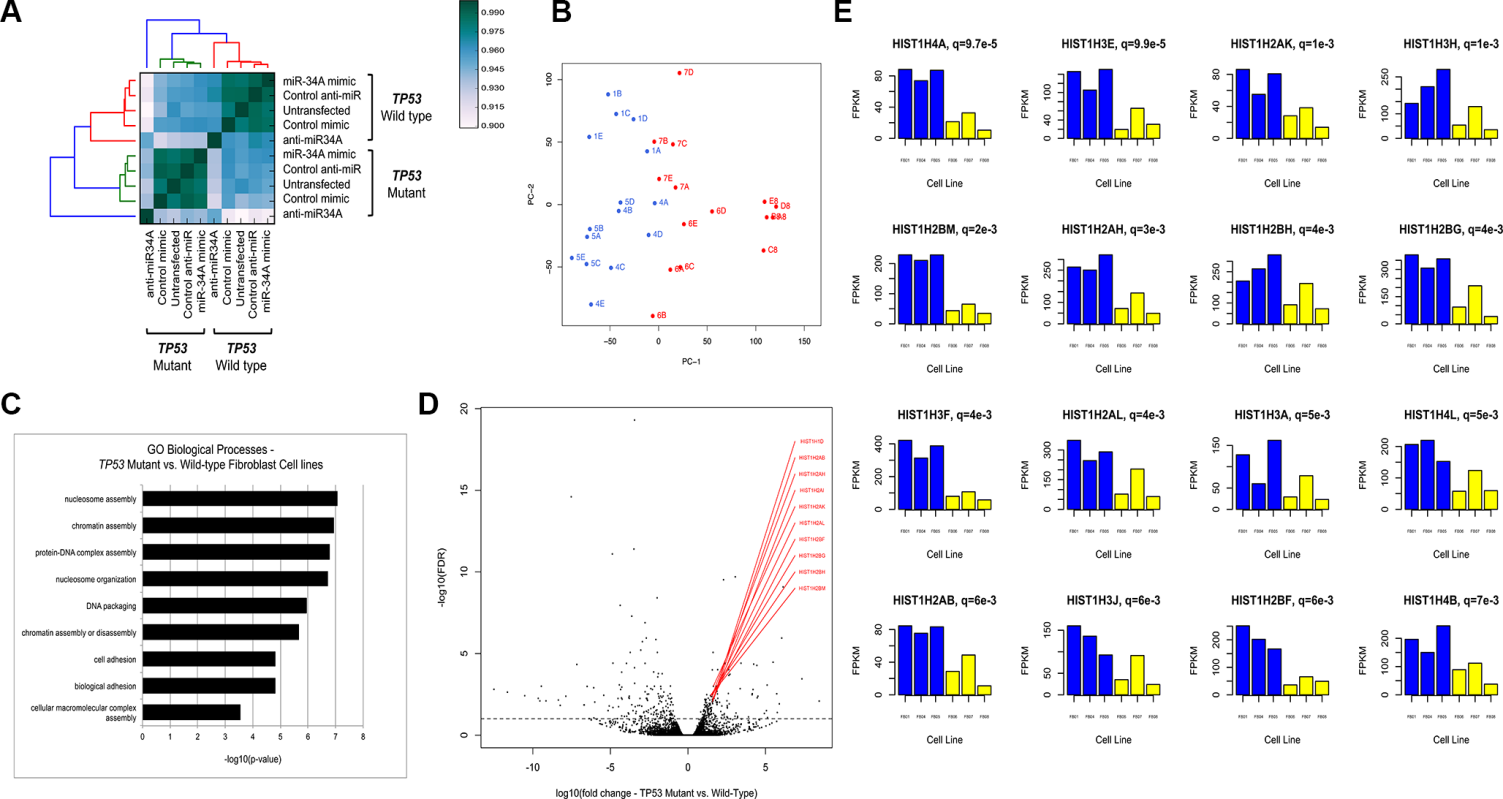
(**A**) Unsupervised hierarchical clustering of pairwise Pearson correlation coefficients of transcripts in RNA-Seq analysis. Pairwise Pearson-correlations were calculated using the log_2_(FPKM) values from the union of all genes that were statistically significant on differential gene expression analysis. (**B**) Principle component analysis of all mapped RNA transcripts depicting the first two principle components (PC-1, PC-2). PC-1 is shown on the horizontal axis, plotted against PC-2 on the vertical axis. *TP53* mutant lines are shown in blue and wild-type lines are shown in red, demonstrating the segregation of *TP53* mutant and wild-type lines in the first principle component. The numbers correspond to the fibroblast cell line ID (A = untransfected; B = control mimic; C = control anti-miR; D = miR- 34A mimic; E = anti-miR-34A). (**C**) GO Biological Processes enriched in transcripts differentially *TP53* mutant fibroblast cell lines relative to *TP53* wild- type lines. The –log10(*p*-value) of enrichment is given on this horizontal axis and GO biological processes are annotated on the vertical axis. (**D**) Volcano plot of differentially expressed RNA transcripts in *TP53* mutant fibroblast cell lines relative to wild-type lines, annotated with histone cluster 1 transcripts. The horizontal axis depicts the log10 fold change transcript expression in *TP53* mutant fibroblast lines relative to wild-type. The vertical axis depicts the negative log10 of the FDR value for each transcript. The genes encoded by the top differentially expressed transcript by lowest FDRvalue are labeled in red. The horizontal dotted line at – log10(FDR) = 1, indicated all points above that line depict transcripts that are differentially expressed at an FDR of < 1%. (**E**) Barplots depicting transcript expression of Histone H1 genes in LFS and *TP53* wild-type fibroblast cell lines. Representative bar plots of the top 16 differentially expressed Histone H1 transcripts in *TP53* mutant fibroblast cell lines relative to wild-type are shown. The horiztonal axis in each plot indicates the cell line ID. *TP53* mutant lines are depicted in blue bars and *TP53* wild-type lines are depicted in yellow. The vertical axis of each plot depicts the FPKM expression values for the respective gene. The q-value between *TP53* mutant and *TP53* wild-type expression for each gene is given, along with the gene name, above each barplot.

Primary skin-derived fibroblast cell lines were generated from 6 pediatric patients who developed malignancies (*TP53* mutant, *n* = 3; *TP53* wild-type, *n* = 3) (Table 1). In order to assess the transcriptional response of these cells to miR-34A modulation, RNA-seq was performed on RNA harvested from untransfected cells as well as cell lines 24 hours post-transfection with hsa-miR-34A-5p mimic or anti-hsa-miR-34a-5p (antagomir), or control oligonucleotides (Table S2). Unsupervised hierarchical clustering on pairwise Pearson correlations of transcript expression values reveals distinct transcriptional signatures segregated by *TP53* mutation status (Figure 1A; Figure S1; Supplementary Data). Moreover, all cell lines transfected with anti-miR-34A differ significantly in their transcriptomic profile relative to all other conditions tested (Figure 1A; Figure S1). Principal component analyses similarly show that the transcriptional profile of cell lines harboring *TP53* mutations are distinct from that of the *TP53* wild-type cells (Figure 1B).

**Table 1:** Mutational and clinical attributes of cell line donors in RNA-Seq experiments

Cell Line ID	Malkin Lab ID	TP53 Mutation	SIFT Classification	Dx	Age (years)
FB01	3506	Exon 5: c.473G > A (p.Arg158His	Deleterious	ADCC	3
FB04	4264	Exon 7: c. 718A > G (p.Ser 240Gly)	Deleterious	CPC	1.6
FB05	4115	Exon 7: c.722C > A (p.Ser241Tyr)	Deleterious	ADCC	10
FB06	3706	Wild-type	-	ADCC	1.5
FB07	3743	Wild-type	-	ERMS-Anaplastic	5
FB08	4059	Wild-type	-	OS	11

* SIFT: amino acid substitution prediction of mutation effect on p53 transactivation function; ADCC = adrenocortical carcinoma; CPC = choroid plexus carcinoma; ERMS = embryonal rhabdomyosarcoma; OS = osteosarcoma; Dx = diagnosis.

The distribution of differentially expressed transcripts is shown in Table S3. There are a significant number of differentially expressed RNA and protein-coding genes within each comparison at a false discovery threshold of 1%, using read mapping and differential transcript detection (EdgeR, Methods). Figure S2 depicts unique and overlapping transcripts for each transfection condition. In untransfected cells, there is a transcriptional pattern that is associated with mutant *TP53* cell lines, consisting of 79 upregulated and 57 downregulated transcripts. NOTCH3 is the most significantly differentially expressed transcript and its expression is decreased in all *TP53* mutant cells, relative to *TP53* wild-type cells (*q* = 4.88e-20, log_10_fold-change =–3.44) (Figure S3).

This transcriptional repertoire was enriched for gene ontology (GO) biological terms affecting epigenetic processes including chromatin dynamics and nucleosome assembly (Figure 1C). In particular, expression of 19 Histone H1 cluster genes is highly increased in the *TP53* mutant fibroblast cell lines relative to wild-type (Figure 1D; Table S5). Analysis of the FPKM expression values for histone cluster 1 genes across all cell lines demonstrates a strong association between *TP53* mutation and elevated relative expression of these genes (Figure 1E).

In the basal state, *TP53* mutations appear to influence the transcriptional landscape of non-transformed cells through a coordinated network of genes affecting epigenetic processes. That high relative expression of histone cluster 1 subunits is strongly associated with *TP53* mutation status suggests that the presence of mutant p53 may influence nucleosome structure and chromatin architecture in this cellular context.

### Transcriptional response of *TP53* mutant and wild-type cells to miR-34A over-expression affects cell cycle mediators and replication-dependent histones

**Figure 2:**
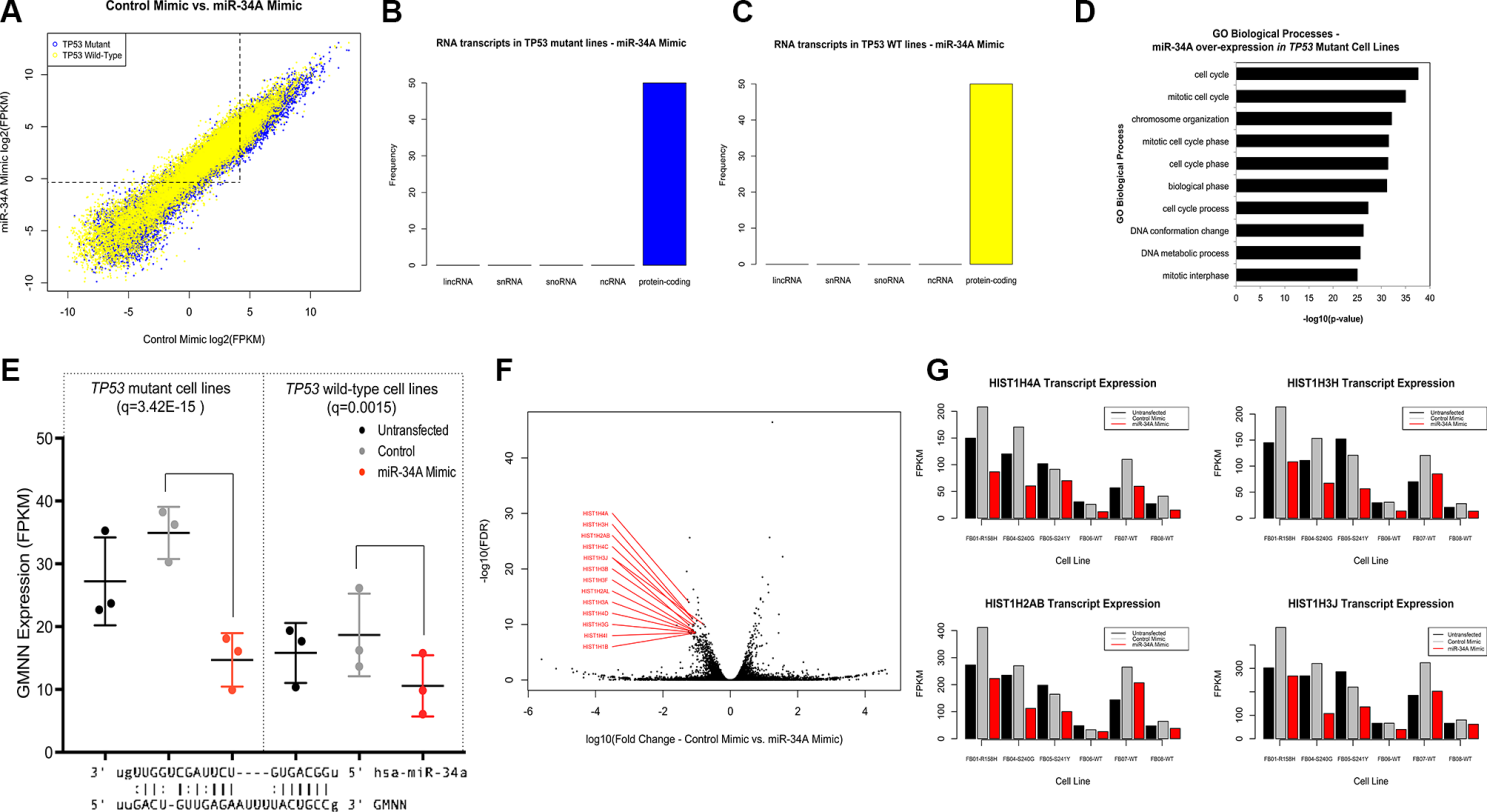
(**A**) Distribution of differentially expressed transcripts in *TP53* mutant and wild-type fibroblast cell lines with elevated miR-34A expression relative to controls. Distribution of differentially expressed transcripts in both *TP53* mutant cell lines (blue) and wild-type cell lines (yellow). Points above and to the left of the dotted line indicate transcripts that are expressed at low levels in cell lines transfected with a control mimic but elevated in cell lines with high relative miR-34A levels. (**B–C**) Distribution of differentially expressed transcripts in (A) *TP53* mutant and (B) wild-type cell lines transfected with miR-34A mimic by RNA classification. Each bar represents a class of RNA (lincRNA: long intergenic non-coding RNA; snRNA: small nuclear RNA; snoRNA: small nucleolar RNA; ncRNA: non-coding RNA, other or uncharacterized). The vertical axis depicts the frequency of this RNA class in the top 50 differentially expressed transcripts in *TP53* mutant lines (blue) and *TP53* wild-type lines (yellow). (**D**) GO Biological Processes enriched in *TP53* mutant fibroblast cell lines transfected with miR-34A mimic. The –log10 (*p*-value) of enrichment is given on this horizontal axis and GO biological processes are annotated on the vertical axis. (**E**) Dot plot of GMNN expression across 3 *TP53* mutant and 3 wild-type cell lines. miR-34A-GMNN base complementarity is given below. The miR-34A seed sequence begins at position 2 from the 5′ end of the transcript. (**F**) volcano plot of differentially expressed Histone H1 transcripts in *TP53* mutant fibroblast cell lines with elevated miR-34A expression. The horizontal axis depicts the log10- transformed fold-change of transcript expression in cells transfected with miR-34A mimic relative to control. The vertical axis depicts the –log10(FDR) values for each transcript. The Histone H1 transcripts with the lowest FDR values are annotated in red. (**G**) Expression of Histone H1 transcripts in *TP53* mutant and wild-type cell lines transfected with elevated miR- 34A expression. Four representative plots are shown for the Histone H1 transcripts with the lowest FDR values. In each plot, the respective cell line per three bars is provided on the horizontal axis, below the cell line transfected with control mimic. Black bars indicate untransfected lines and red bars indicate cell lines transfected with miR-34A mimic. The vertical axis depicts the FPKM expression value for each transcript across all cell lines at each transfection condition.

The transcriptional profile of cells transfected with hsa-miR-34A-5p mimic was systematically assessed in order to determine the impact of ectopic miR-34A expression in *TP53-*mutant and wild-type cell lines (Figure 2A). The top 50 significantly differentially expressed transcripts in both *TP53* mutant lines and wild-type lines transfected with miR-34A mimic were exclusively protein-coding (Figure 2B–2C). Distinct transcriptional patterns were observed in cells with elevated miR-34A expression relative to controls in both *TP53* mutant and wild-type cell lines (Figure S4). A total of 128 transcripts were commonly differentially expressed in *TP53* mutant and *TP53* wild-type cell lines transfected with the miR-34A mimic (Table S6). This set of transcripts is enriched for genes encoding proteins involved in cellular processes including cellular organization and mitotic cell cycle (Figure S5). E2F1 – a known miR-34A target gene [12] - is down-regulated in response to miR-34A mimic transfection to a similar extent in *TP53* mutant lines and *TP53* wild-type lines (*TP53* mutant: log_10_fold-change = −0.83, *q* = 9.2e-6; *TP53* wild-type: log_10_fold-change = −0.85, *q* = 5.5e-4). Similarly, miR-34A is known to influence expression of CDK4 and this transcript is down-regulated in both *TP53* mutant and *TP53* wildtype cell lines (*TP53* mutant: log_10_fold-change= −0.86, *q* = 8.3e- 12; *TP53* wild-type: log_10_fold-change= −0.69, *q* = 9.4e-5). These common differentially expressed transcripts may represent cellular effects of elevated miR- 34A expression independent of *TP53* mutation status and/or variable effect of transcriptional repression or induction in relation to *TP53* mutation status.

GO term enrichment analysis of the miR-34A-regulated transcripts in the *TP53* mutant cell lines demonstrates that genes are strongly enriched for cell cycle mediators (Figure 2D). To assess the extent to which miR-34A may regulate these transcripts, through direct seed sequence complementarity, the most statistically significant transcripts (*p* < 0.001) that were down-regulated secondary to miR-34A over-expression (n_TP53 mutant_= 277, n_TP53 wild-type_=17) were surveyed for the ACTGCC sequence (miR-34A, 3′ugUUGGUCGAUUCUGUGACGGu 5′ – miR-34A) (Tables S7–S8). Among the most highly downregulated transcripts containing this sequence, include Geminin, encoded by *GMNN* at the 6p22 locus (log_10_fold-change = −1.27; *q* = 3.42e-15). Down-regulation of GMNN is observed in response to ectopic miR-34A expression in *TP53* mutant cell lines, and to a lesser extent in wild-type cell lines (Figure 2E). GMNN has been shown to serve as a master regulator of cell cycle progression that controls onset of DNA replication and prevents re-replication [13].

Intriguingly, transcript expression of 6p22 histone cluster 1 transcripts that are otherwise elevated in *TP53* mutant cells (as shown in Figure 1E), was significantly decreased in *TP53* mutant lines transfected with miR-34A mimic, but were essentially unaffected in wild-type cell lines (Figure 2F). This unanticipated finding may suggest that high miR- 34A expression suppresses histone cluster 1 genes and this effect is dependent on defective or haploinsufficient p53 (Figure 2G). Histone cluster 1 genes containing miR- 34A seed complimentary sequence include HIST1H4A (1.02e- 14), HIST1H3J (*q* = 1.18e-10) and HIST1H1A (*q* = 4.07e-6).

In summary, among the repertoire of differentially expressed RNA transcripts, there is a prominent effect of elevated miR-34A expression on critical cell cycle mediators, including regulation of replication-associated transcripts at the 6p22 locus, in the context of mutant *TP53*.

### Transcriptional response of *TP53* mutant and wild-type cells to miR-34A inhibition is characterized by changes in non-coding RNA transcripts

**Figure 3:**
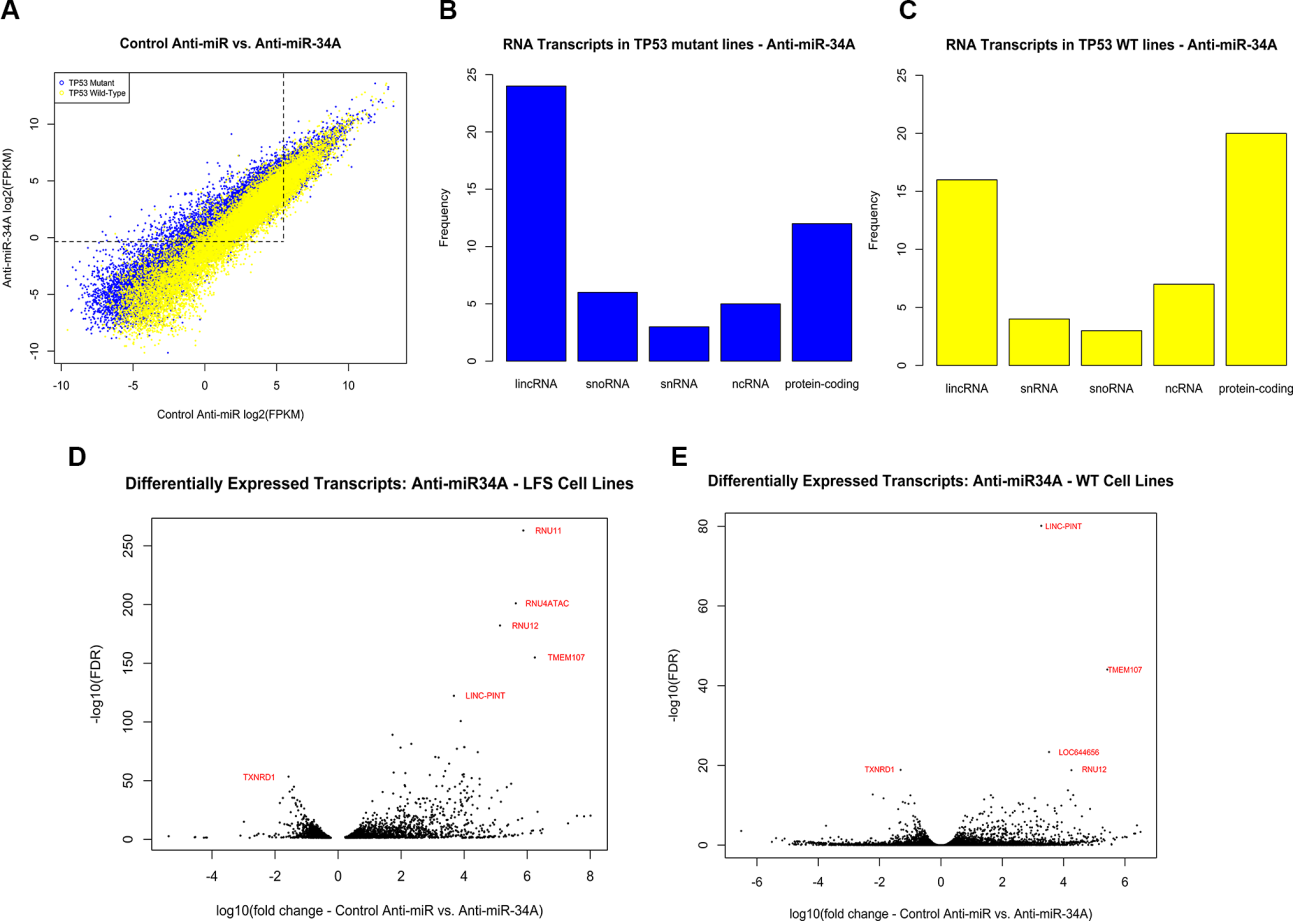
(**A**) Distribution of differentially expressed transcripts in both TP53 mutant cell lines (blue) and wild-type cell lines (yellow). Points above and to the left of the dotted line indicate transcripts that are expressed at low levels in cell lines transfected with a control anti-miR but elevated in cell lines with low relative miR-34A levels. (**B–C**) Distribution of differentially expressed transcripts in (A) TP53 mutant and (B) wild-type cell lines transfected with anti-miR-34A by RNA classification. Each bar represents a class of RNA (lincRNA: long intergenic non-coding RNA; snRNA: small nuclear RNA; snoRNA: small nucleolar RNA; ncRNA: non-coding RNA, other or uncharacterized). The vertical axis depicts the frequency of this RNA class in the top 50 differentially expressed transcripts in *TP53* mutant lines (blue) and *TP53* wild-type lines (yellow). (**D–E**) Volcano plots of differentially expressed transcripts in (A) *TP53* mutant and (B) wild-type fibroblast cell lines with diminished miR-34A expression. The horizontal axis depicts the log10-transformed fold-change of transcript expression in cells transfected with anti-miR-34A relative to control. The vertical axis depicts the –log10(FDR) values for each transcript. The transcripts with the lowest FDR values are annotated in red.

The number of differentially expressed transcripts detected in the presence of miR-34A inhibition is higher than in the condition of miR-34A over-expression in both wild-type and mutant lines (Figure S2), indicating that loss of miR-34A may have a more widespread impact on the transcriptome in this cellular context. The transcriptional response to miR-34A inhibition by hsa-anti-miR-34A- 5p oligonucleotides in *TP53* mutant and *TP53* wild-type non-transformed cells revealed a distinct pattern of *TP53* mutation-associated signatures, with a prominent effect of elevated transcript expression in *TP53* mutant cells (Figure 3A). Indeed, the number of differentially expressed transcripts in *TP53* mutant cells transfected with anti-miR-34A relative to control was much higher than that of *TP53* wild-type cells (2631 transcripts and 357 transcripts, respectively), however, 91% (324/357) of transcripts found differentially expressed in wild-type cells were also differentially expressed in the *TP53* mutant lines (Figure S2). Unlike non-transformed cells with elevated miR-34A expression, inhibition of miR- 34A results in significant deregulation of non-coding RNA transcripts (Figure 3B–3C). Among the most significantly differentially expressed transcripts in both *TP53* mutant and wild-type cells transfected with anti-miR-34A are numerous long intergenic non-coding RNAs (lincRNAs), other noncoding RNAs, as well as protein-coding transcripts that have been linked to *TP53* and miR-34A regulation, such as SLC7A11 and PPP1R10, respectively [14, 15] (Figures S6–S7). Interestingly, suppression of miR- 34A also leads to significantly decreased TP53 transcript expression in *TP53* mutant, this effect is not significant in wild-type cell lines (Figure S8). There was no effect on TP53 transcript levels following miR-34A over-expression.

**Figure 4:**
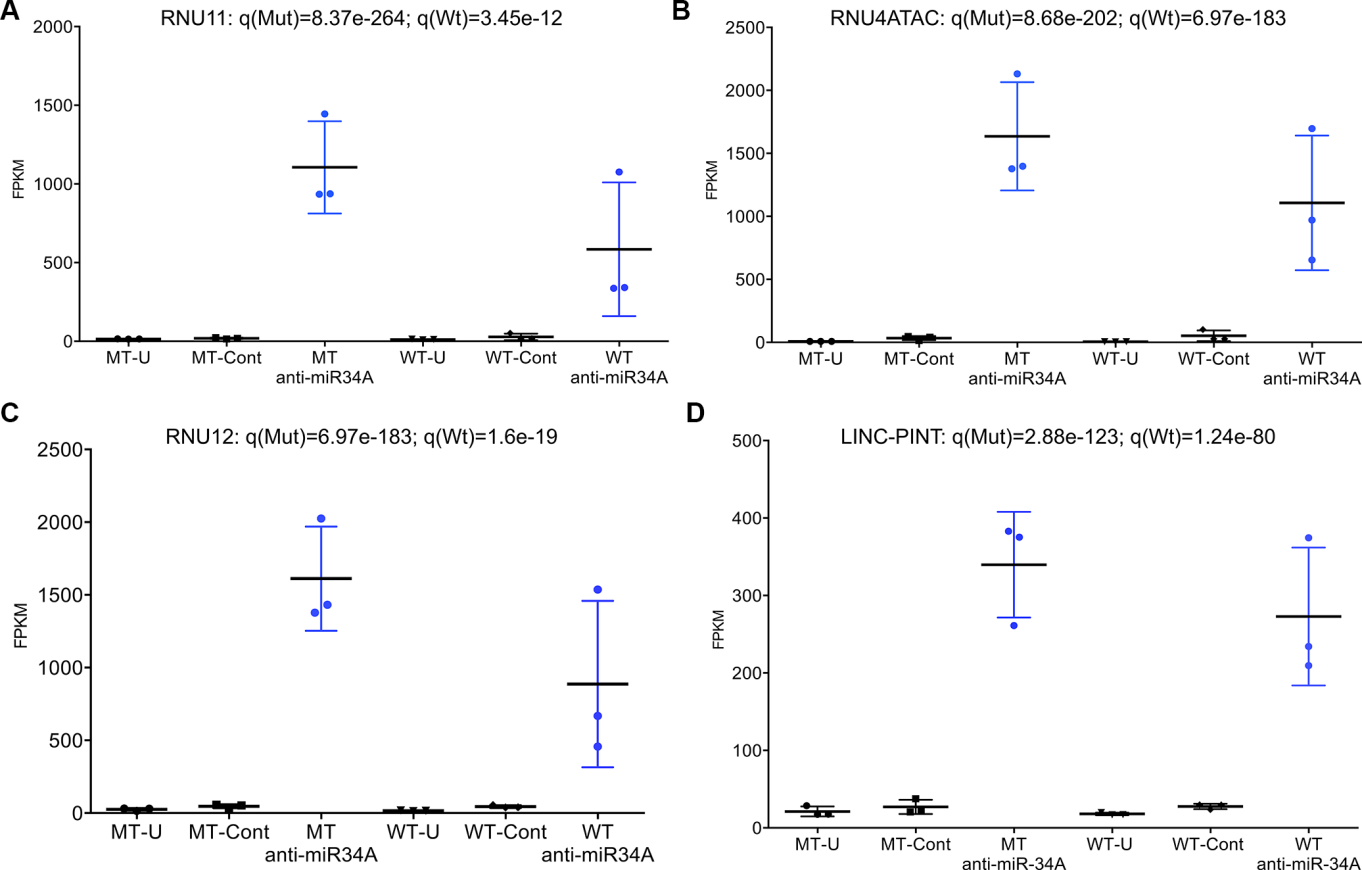
(**A**–**D**) Dot plots of most significantly differentially expressed transcripts in *TP53* mutant cell lines (*n* = 3) and *TP53* wild-type cell lines (*n* = 3). MT = mutant; WT = wild-type; U = untransfected; Cont = control; FPKM = fragments per kilobase of transcript per million mapped reads (expression level). Significance measures adjusted for false discovery rate (*q*-value) are given for each transcript across *TP53* mutant and *TP53* wild-type cell lines.

Three key components of the minor (U12) spliceosome are among the most highly differentially expressed transcripts in *TP53* mutant cells transfected with anti-miR-34A. These transcripts are constituents of a larger complex that catalyzes the removal of an atypical class of spliceosomal introns (U12) from mRNA transcripts. Although U12-type introns comprise a minor fraction of all introns in human cells, they occur in genes essential for cell viability [16]. The minor spliceosome consists of U11, U12, U4atac, U6atac and U5 [16]. U11, U12 and U4atac are all found to be significantly upregulated to similar levels in cells transfected with anti-miR-34A (Figure 3D–3E; Figure 4A–4C; RNU11: q(Mut) = 8.37e-264, q(Wt) = 3.45e-12; RNU4ATAC: q(Mut) = 8.68e-202, q(Wt) = 6.97e-183; RNU12: q(Mut) = 6.97e-183; q(Wt) = 1.6e-19). This effect is more pronounced in some *TP53* mutant cell lines relative to wild-type cell lines.

The mature RNA transcripts of these components of the U12 spliceosome do not harbor miR-34A complimentary seed sequences, suggesting either miR- 34A regulates these transcripts indirectly through other transcripts or through global effects on the cell cycle state. To further validate that this effect occurs in response to miR-34A inhibition, despite the absence of putative direct base pairing, transcript levels of one of the U12 spliceosome components (RNU4ATAC) were first validated by qPCR, then replicated in two *TP53* mutant cell lines with increasing doses of anti-miR-34A (FB04 [S240G] cell line utilized in RNA-Seq experiment, and an independent cell line harboring a R248Q mutation) (Figures S9–S11).

Finally, suppression of miR-34A expression was found to result in widespread up-regulation of long intergenic non-coding RNA transcripts in association with p53 mutation status (Tables S9–S10). In *TP53* wild-type cell lines transfected with anti-miR-34A, the most significantly differentially expressed transcript is LINC-PINT (long intergenic non-coding RNA, p53-induced transcript) (*q* = 1.24e-80) (Figure 4D). It is also among the most highly induced transcripts in *TP53* mutant lines transfected with anti-miR-34A (*q* = 2.88e-123) (Figure 4D). LINC-PINT was first identified as a ubiquitously expressed lincRNA that is regulated by p53 and its over-expression has been shown to inhibit proliferation of tumor cells [17]. Although this transcript does not contain a sequence complimentary to the miR-34A seed region, miR-34A inhibition is directly correlated with LINC-PINT expression with increasing doses of anti-miR-34A (Figures S12–S13). In a non-transformed cellular context, inhibition of miR- 34A strongly induces LINC-PINT across both *TP53*-mutant and *TP53* wild-type cell lines, suggesting that miR- 34A may regulate expression of this lincRNA, independent of p53.

These findings demonstrate that, under basal conditions, expression of non-coding RNAs such as minor spliceosome components and LINC-PINT is maintained at low levels and inhibition of miR-34A leads to marked de-repression of these targets, among other non-coding transcripts.

### miR-34A is essential for cell viability and repression of miR-34A results in G2/M cell cycle arrest

**Figure 5:**
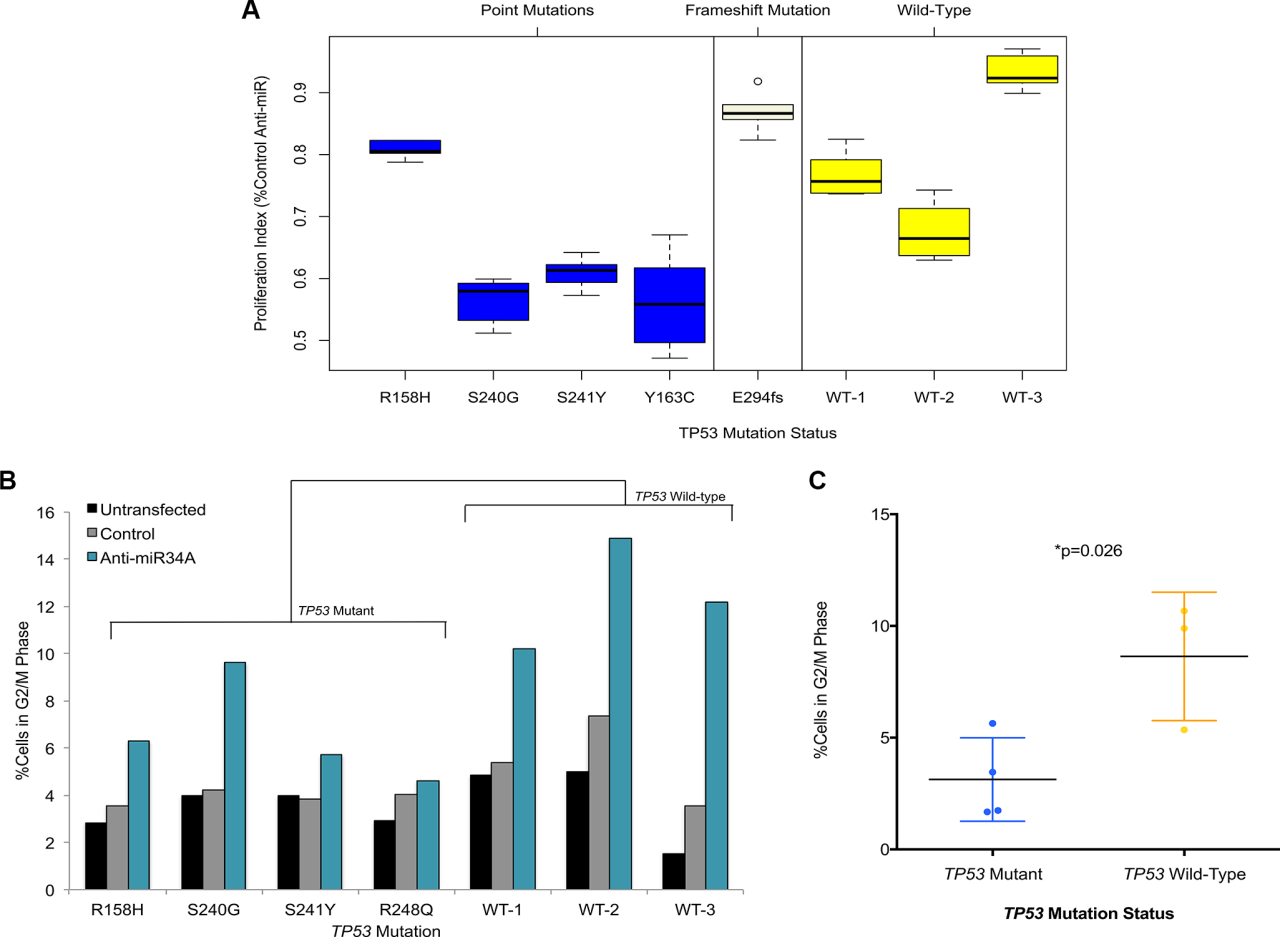
(**A**) Box plots of anti-miR-34A proliferation assays in *TP53* mutant and *TP53* wild-type primary fibroblast lines. The vertical axis depicts the proliferation index for the respective cell line transfected with an anti-miR-34A oligonucleotide, normalized to untransfected cells and given as a fraction of the cell proliferation index of the same cell line transfected with a control anti-miR oligonucleotide. The WT lines are annotated as follows: WT-1 (FB06), WT-2 (FB07), WT-3 (FB08). Data is shown for six technical replicates and each experiment was performed in at least one additional biological replicate. (**B**) Proportion of cells in G2/M phase determined by EdU/propidium iodide staining and flow cytometry. *TP53* mutation status is provided on the horizontal axis and proportion of cells in G2/M is given on the vertical axis. *P*-value determined by two-tailed *t*-test comparing mean differences across *TP53* mutant cell lines relative to *TP53* wild-type cell lines. (**C**) Dot plots of fraction of cells in G2M following miR-34A inhibition in *TP53* mutant cell lines (*n* = 4) and TP53 wild-type cell lines (*n* = 3). Fraction (%) was determined by comparing the percent of cells in G2M for each cell line in the following miR-34A inhibition relative to untransfected cells for each respective cell line. *P*-value determined by two-tailed *t*-test comparing mean differences across *TP53* mutant cell lines relative to *TP53* wild-type cell lines.

To functionally characterize the phenotypic impact of miR-34A on non-transformed cells, we sought to determine the effects of miR-34A suppression on cell viability. Suppression of miR-34A using an anti-miR- 34A oligonucleotide reproducibly diminishes the cell proliferation rate of all three *TP53* mutant cell lines used in the transcriptome assays, but to a lesser extent in the *TP53* wild-type lines (Figure 5A). This decrease in cell proliferation was not found to be due to increased activation of canonical apoptosis pathways (Figure S14). To further validate this finding, two additional cell lines harboring *TP53* mutations, FB02 (E294fs) and FB03 (Y163C) were transfected with the anti-miR-34A oligonucleotide. FB02 harbors a mutation that results in a frameshift of the open reading frame of p53, and likely does not lead to a viable protein product. Accordingly, there was no significant effect on suppression of cell proliferation mediated by miR-34A inhibition in this cell line (Figure 5A). By contrast, inhibition of miR-34A in FB03, harboring a non-synonymous mutation at codon 163, resulted in decreased cell proliferation, as detected in the initial *TP53* mutant cell lines studied (Figure 5A).

However, within the *TP53* mutant cell lines harboring point mutations, the effects of inhibition of anti-miR-34A on the *TP53* R158H cell line is less pronounced than the effect observed on the other LFS lines. This may be due to the relative haploinsufficiency of p53 in the other *TP53* mutant fibroblast cell lines, relative to the effect imparted by this mutation.

Finally, cell cycle analysis reveals that suppression of miR-34A results in a shift in a fraction of the cell population into G2/M phase relative to untransfected cells (Figure 5B; Figure S15A–S15B). This effect is less pronounced in *TP53* mutant cells in comparison to wild-type cells (Figure 5C). These findings corroborate the transcriptomic results that demonstrate miR-34A-associated regulation of essential cell cycle mediators.

## DISCUSSION

This study describes the first analysis of the miR-34A-p53 axis in a non-transformed cellular context and provides insight into the underlying transcriptional networks in association with mutant *TP53* and miR-34A. Modulating miR-34A in cell lines from patients harboring germline *TP53* mutations provides a unique approach to characterizing this network. The transcriptional response to miR-34A modulation reveals that this miRNA may be a crucial switch that can impact expression of numerous cell cycle regulators and non-coding RNA networks. The effect of miR-34A modulation on cell cycle regulatory genes is consistent with previous reports of miR-34A regulated transcripts in other cellular contexts [10]. In addition to p53-mediated pathways, miR-34A is found to regulate other novel pathways, as evidenced by the differential effect of miR-34A transcriptional landscape in the context of mutant p53. Previous studies have shown that human cell lines with miR-34A knockouts have unimpaired p53-mediated responses to genotoxic stress and miR-34A is therefore dispensible for the p53-mediated response to stress in human cells [4]. Similarly, our findings demonstrate that miR-34A and p53 have overlapping but also autonomous roles, and underscore the importance of this microRNA as a key component of p53-dependent and p53-independent cellular pathways.

The majority of *TP53* mutation-associated transcripts, under basal conditions, are involved in chromatin remodeling and nucleosome assembly. Transcripts associated with mutant *TP53* include the 6p22 histone cluster 1 genes. H1 histones are referred to as linker histones and bind to nucleosome constituents, stabilizing their interaction with DNA. Studies in H1-depleted mouse embryonic fibroblasts have shown that cells require homeostatic H1 levels to maintain their epigenetic landscape [18]. That exogenous miR-34A expression suppresses many of these replication-dependent transcripts in the presence of *TP53* mutations, and to a greater extent than wild-type *TP53*, suggests that miR- 34A may play an important role in sustaining epigenetic integrity when p53 function is compromised by mutation.

Suppression of miR-34A strongly de-represses many non-coding RNA transcripts. Marked induction of key components of the minor (U12) spliceosome occurs in response to miR-34A suppression. Studies in zebrafish have demonstrated a conserved role for minor splicing in cell cycle progression [19]. These findings point to a novel putative role of miR-34A in modulating transcription of the U12 spliceosomal machinery, likely indirectly through its impact on the cell cycle. miR-34A is also is associated with transcriptional repression of a host of lincRNAs, including LINC-PINT, a p53-induced lincRNA [17]. LincRNAs show strong evolutionary conservation and accordingly, were hypothesized to be essential in a number of cellular processes including cell cycle regulation [20]. LincRNAs can also physically associate with repressive chromatin modifying complexes suggesting a role for these non-coding RNAs in transcriptional regulation [21]. Our findings are the first to identify miR-34A as an important node in the transcriptional regulation of numerous lincRNAs and point to further study of micro-RNA-lincRNA related pathways.

Lastly, miR-34A suppression results in an increased fraction of cells arrested in the G2/M phase. It is well known that DNA damage induces a p53 response, involving coordinated repression of genes encoding proteins required for G2 and M phase, and loss of essential cell cycle proteins arrests cells in the G2 phase [22]. Since the G2 checkpoint prevents cells from entering mitosis when DNA is damaged, suppression of miR-34A resulting in G2/M arrest demonstrates that this microRNA is indeed essential to cell cycle progression, and this response may be diminished in the absence of functional p53. Our results suggest that miR-34A, much like p53, is an essential cell cycle mediator and future studies aimed at further characterizing the phenotypic impact of miR-34A suppression using alternate approaches may be warranted.

## MATERIALS AND METHODS

### Patient-derived cell lines and ethics approval

Immortalized skin fibroblasts derived from patients harboring germline *TP53* mutations and *TP53* wild-type patients (from TCAG Biobanking Facility, Toronto, CA) were cultured in Alpha Modification of Eagle's Medium (AMEM; Wisent Inc.) supplemented with 10% fetal bovine serum (Wisent Inc). For the use of primary patient samples for this research, the study was approved by the Hospital for Sick Children Research Ethics Board under the study title “Molecular characterization of Li-Fraumeni Syndrome and its variants” (REB File No.: 0019910602).

### miR-34A mimic and anti-miR transfections

Fibroblast cell lines were cultured in Alpha Modification of Eagle's Medium (AMEM; Wisent Inc.) supplemented with 10% fetal bovine serum (Wisent Inc.). Briefly, 200,000 (confluent) cells were seeded into a well of a 6-well plate 24 hours before transfection with 3 ul of Lipofectamine 2000 (Invitrogen) and 75 nM of hsa-miR- 34a-5p/control mirVana mimics or hsa-anti-miR-34a-5p/control mirVana inhibitors (Ambion, Life Technologies). Transfected cells were maintained at 37°C in a humidified 5% CO2 incubator until assay use. Transfection efficiency was validated by concomitant transfection with GFP pMAX fluorescent reporter, as well as qPCR validation in three cell lines to test miR-34A over-expression post-transfection (Figure S16). qPCR validation of anti-miR-mediated suppression of miR-34A expression was also performed (Figure S17). miR-34A target transcripts that were validated by Western blot and/or reporter assays were downloaded from the miRTarBase database (http://mirtarbase.mbc.nctu.edu.tw/), and expression of these transcripts was determined in the anti-miR-34A RNA-Seq dataset (described below). A number of known miR-34A target transcripts were significantly de-repressed in cells transfected with the hsa-miR-34A-5p inhibitor providing further support for the efficacy of this reagent in targeting miR-34A (Figure S18, Table S11).

### RNA extraction and RNA-sequencing

RNA was extracted from fibroblast cell lines transfected with miR-34A mimic or anti-miR or respective controls, 24 hours post-transfection, using standard protocols. Stranded mRNA library preparation was performed for next generation paired-end sequencing using the Illumina HiSeq2500 platform. Reads were aligned to human genome reference sequence, build hg19 (RefSeq gene models). The quality of the sequence data was assessed using FastQC v.0.11.2 (http://www.bioinformatics.babraham.ac.uk/projects/fastqc/). Adaptors were trimmed using using Trimmomatic v0.32 (http://www.usadellab.org/cms/?page=trimmomatic). Trimmomatic is run with the following parameters: ILLUMINACLIP:TruSeq3-PE-2.fa:2:30:10 SLIDINGWINDOW:4:20 MINLEN:20. Based on the above, the trimming is performed in the following order: (1) ILLUMINACLIP Trims Illumina adaptors; allowing maximum 2 mismatches for a full match to be performed; 30 is the palindromeClipThreshold, which specifies how accurate the match between two ‘adapter ligated' reads must be for paired end palindrome read alignment; 10 is simpleClipThreshod, which specified how accurate the match between any adapter etc. sequence must be against a read (for more detailed explanation on how this score is calculated please see trimmomatic manual); (2) SLIDINGWINDOW:4:20 specifies average quality of 20 required over 4 bases, lower quality ends are trimmed; (3) MINLEN 20 specifies minimum length of 20 bases for reads to be kept. The quality of the trimmed reads is re-assessed with FastQC. The trimmed reads were also screened for presence of rRNA and mtRNA/DNA sequences using FastQ-Screen v.0.4.3 (http://www.bioinformatics.babraham.ac.uk/projects/fastq_screen/). Prior to trimming, there was an average of 24 038 766 paired reads per sample and 19 636 960 reads following trimming. The raw trimmed reads were aligned to the reference genome (hg19) using Tophat v. 2.0.11 (http://ccb.jhu.edu/software/tophat/index.shtml). Tophat alignments are processed to extract raw read counts for genes using htseq-count v.0.6.1p2(HTSeq, http://www-huber.embl.de/users/anders/HTSeq/doc/overview.html). Assigning reads to genes by htseq-count was performed in the mode “intersection_nonempty”, i.e. if a read overlaps with two overlapping genes and the overlap to gene A is greater than the overlap to gene B, the read is counted towards gene A, while if a read overlaps equally with gene A and gene B, then it is not counted towards either gene.

### Differential expression analysis

Raw gene counts were loaded and sample-normalized using DESeq v.1.18.0 (http://bioconductor.org/packages/release/bioc/html/DESeq.html). In the filtered data set we have retained only genes whose cpm (counts per million reads) is > 0.25 in at least two samples. PCA (Principal Component Analysis) is performed to assess relation among samples; an empirical permutation-based procedure is used to identify informative principal components. Two-condition differential expression was performed using the EdgeR R package, v.3.8.6 (www.bioconductor.org/packages/release/bioc/html/edgeR.html).

Unpaired or paired design was used, as described in the Analysis objectives. Paired design accounts for samples originating from the same patients. Genes were discarded if their maximum sample-normalized count across samples is lower than the 25% quantile of max sample-normalized counts (this is intended to remove genes that are not expressed). For all comparisons the method used for normalizing the data was TMM, implemented by the calcNormFactors(y) function. The dispersion estimation was produced by the method estimateGLMCommonDisp(y, design). FPKM values were used to generate all barplots using the R statistical package. Using an orthogonal approach to sequence mapping and differential transcript detection (Cufflinks), similar patterns of differential transcript abundance were detected at a false discovery rate threshold of 5% (Table S4). Cufflinks employs more stringent criteria for FDR imputation and accordingly, the threshold for determine significantly differentially expressed transcript was more liberal than that set for the EdgeR-derived values. FPKM values derived from the Cufflinks algorithm were used to generate the Pair-wise Pearson correlation heatmap.

### Cell viability assays

Twenty-four hours following miRNA transfection, 5,000 fibroblast cells were seeded into a 96-well plate, and incubated in a humidified 5% CO2 incubator for 96 hours. To assess cellulat viability, 20 ul of the MTS reagent (Promega) was added to each well and a spectrophotometer (490 nm) was used to measure the resulting absorbance changes. All absorbance values were adjusted by subtracting the background absorbance of empty wells. Results were normalized to control-transfected cells and represent the mean of three biological replicates.

### Apoptosis assay

Apoptotic cells were identified using phycoerythrin (PE)-conjugated anti-annexin V antibody and 7-aminoactinomycin D (7AAD) dual stain following the manufacturer's instructions (BD Pharmingen). Briefly, fibroblast cells were washed twice in cold PBS and resuspended in 1 × binding buffer (BD Pharmingen) at a concentration of 1×10^6^ cells/mL, 48 h after miR-34A mimic/anti-miR oligonucleotide transfection. 100 μL of cell suspension was transferred to a conical tube and 5 μL each of annexin V-FITC and 7AAD were added. Cells were gently vortexed and incubated for 15 min at room temperature in the dark. 400 μL of 1 × binding buffer was added to each sample before analysis by fluorescence activated cell sorting (BD FACSCalibur). For each sample, 10,000 events were acquired and experiments were performed two separate times in triplicate. Bar graphs depict Annexin V/7AAD positive fraction.

### EdU-propidium iodide staining and flow cytometry

Cells were transfected with anti-miR-34A oligonucleotide or control, as described above. Cells were incubated with 10 uM EdU (Thermo Fisher, MA, USA) in culture medium for 2 hours and subsequently harvested. Cells were washed once with 3 mL of 1% BSA in PBS, pelleted and dislodged in 100 uL of Click-iT fixative (Thermo Fisher) and incubated for 15 minutes at room temperature, protected from light. Cells were again washed with 1% BSA in PBS, pelleted and dislodged in 100 uL of saponin-based wash reagent. Cells were subsequently stained with Alexa Fluor 647 fluorscent dye azide and incubated for 30 minutes at room temperature. Cells were then washed with 3 mL of saponing-based wash buffer, pelleted, and resuspended in 500 uL 2 mg/mL RNAse A. Following a 5 minute incubation, 500 uL of 0.1 mg/mL propidium iodide solution (DNA stain) was added and incubated at room temperature. The solution was transferred to a filter-cap round-bottom tube and EdU and PI acquisition was performed by flow cytometry with 633/635 nm excitiation with a red emission filter. Cell population fractions were determined by the proportion of cells in each quadrant of the FACS plot.

## CONCLUSIONS

Collectively, the findings from this study support a putative role for miR-34A in acting as an essential regulator of broad transcriptional networks that converge on the cell cycle. Through its complementary and redundant roles to p53, miR-34A may compensate for the effects of mutant p53 in non-transformed cells. This study unravels a new layer of complexity in the miR-34A pathway, demonstrating that the impact of miR-34A modulation involves a coordinated network of lincRNAs, RNA components of the U12-dependent spliceosome and protein-coding histone genes that are essential for cellular maintenance. Aberrations in any of these pathways may represent vulnerabilities to tumor development. These studies provide strong insight into the tumor suppressive capacity of miR-34A, demonstrating why miR-34A inactivation may be pervasive in human malignancies. The identification of novel coding and non-coding miR- 34A targets provides new avenues for intervention of the cellular pathways deregulated by miR-34A in human cancers.

## SUPPLEMENTARY MATERIALS FIGURES AND TABLES




