# Supplementary material for: Transcriptome-wide characterization of the endogenous miR-34A-p53 tumor suppressor network
Source: Oncotarget. 2016 Jul 6;7(31):49611–22. doi: 10.18632/oncotarget.10417 (PMC5226533; doi:10.18632/oncotarget.10417)
Supplement: Supplementary file 1 [file oncotarget-07-49611-s001.pdf]

Transcriptome-wide characterization of the endogenous miR-34A-p53 tumor suppressor network

Supplementary Materials

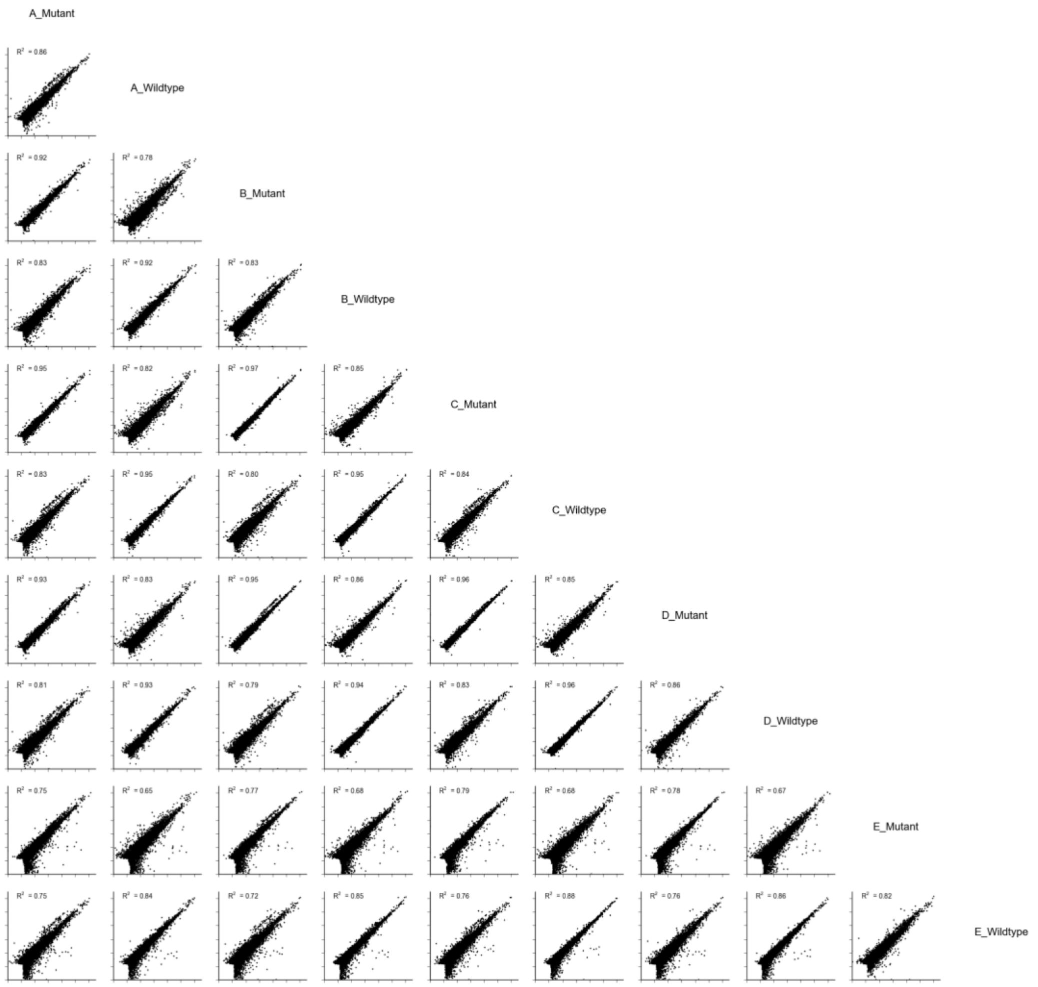

Supplementary Figure S1: Pearson Correlation (PC) scatter plots. Scatter plots depicting log2(FPKM + 1) values between sample pairs (transfection conditions) in the RNA-Seq dataset and the Pearson Correlation coefficient (A: untransfected; B: control mimic; C; control anti-miR; D: miR-34A mimic; E: anti-miR-34A). Values from each individual plot contributed to the Pearson correlation heatmap in Figure 1A of the manuscript.

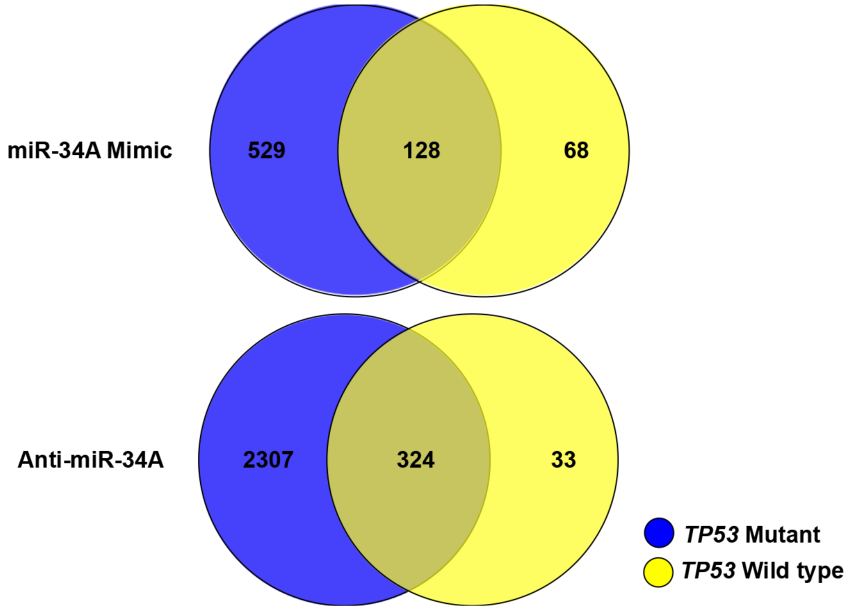

Supplementary Figure S2: Unique and overlapping differentially expression transcripts in miR-34A over-expressing cells and miR-34A-deficient cells – *TP53* mutant and wild-type cell lines. The Venn Diagrams depict the differentially expressed transcripts determined by the EdgeR algorithm in cells transfected with miR-34A mimic relative to control (top diagram) and cells transfected with anti-miR-34A relative to control (bottom diagram).

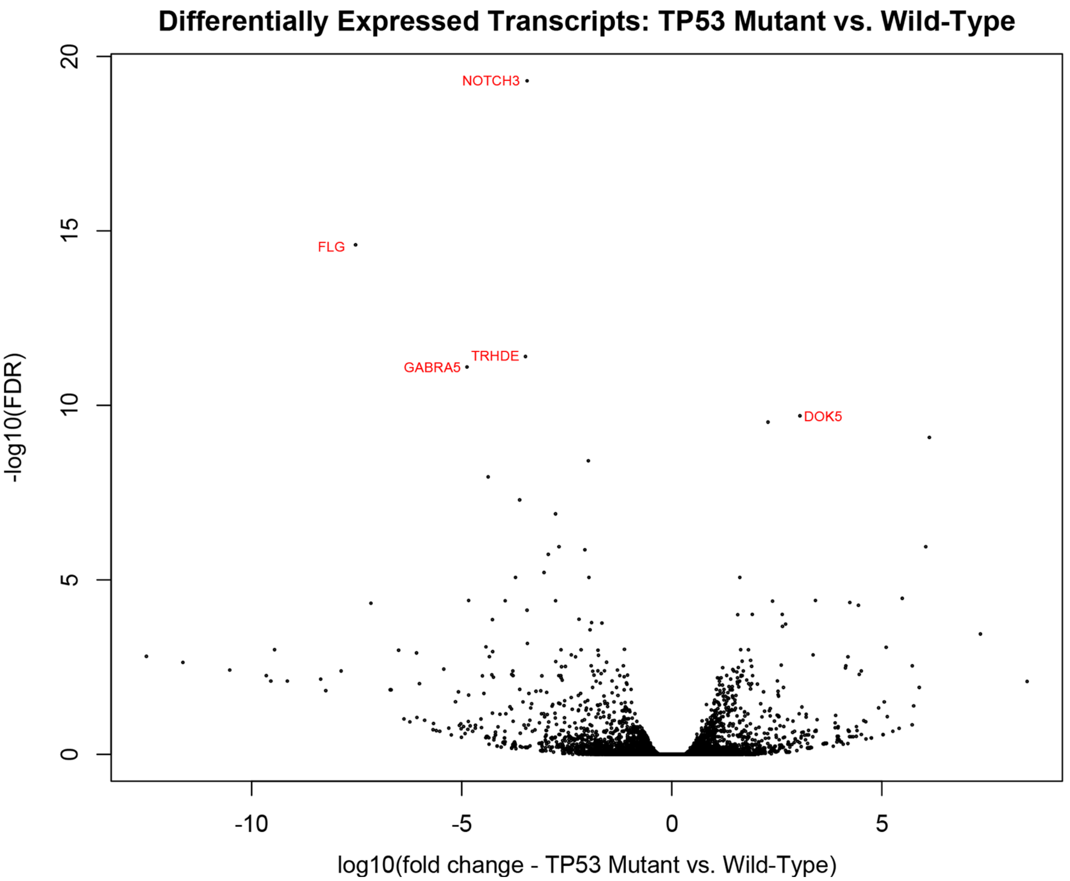

Supplementary Figure S3: Volcano plot of differentially expressed RNA transcripts in *TP53* mutant fibroblast cell lines relative to wild-type lines. The horiztonal axis depicts the log10 fold change transcript expression in *TP53* mutant fibroblast lines relative to wild-type. The vertical axis depicts the negative log10 of the FDR value for each transcript. The genes encoded by the top differentially expressed transcript by lowest FDR value are labeled in red.

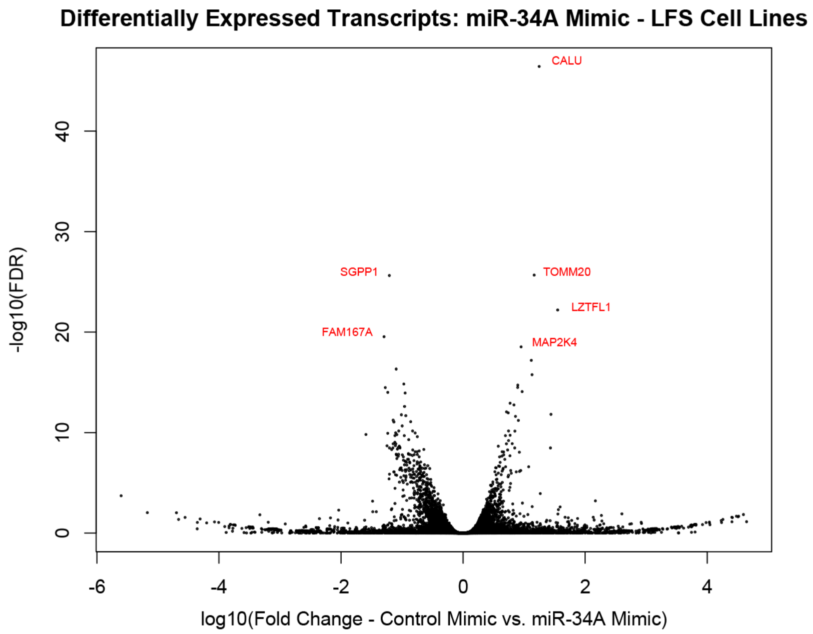

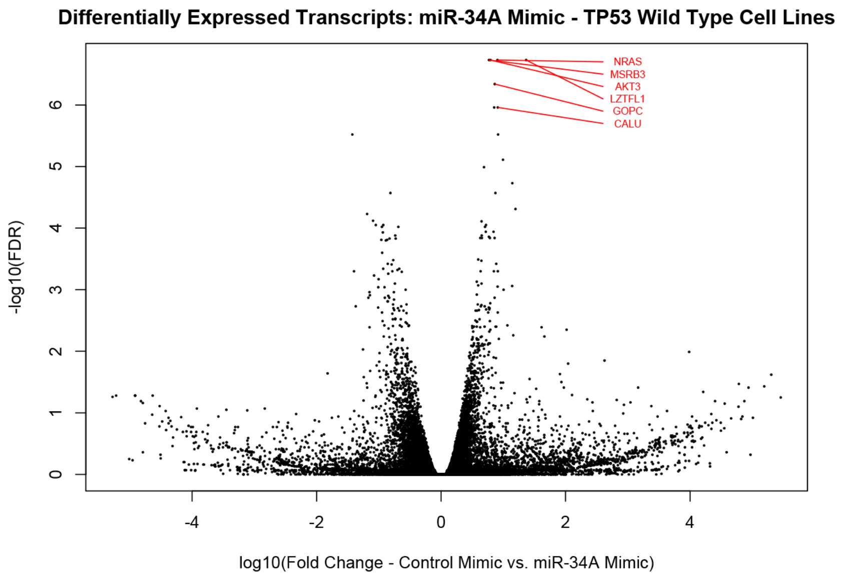

Supplementary Figure S4: (**A**–**B**) Volcano plots of differentially expressed transcripts in (A) *TP53* mutant and (B) wild-type fibroblast cell lines with elevated miR-34A expression. The horizontal axis depicts the log10-transformed fold-change of transcript expression in cells transfected with miR-34A mimic relative to control. The vertical axis depicts the –log10(FDR) values for each transcript. The transcripts with the lowest FDR values are annotated in red.

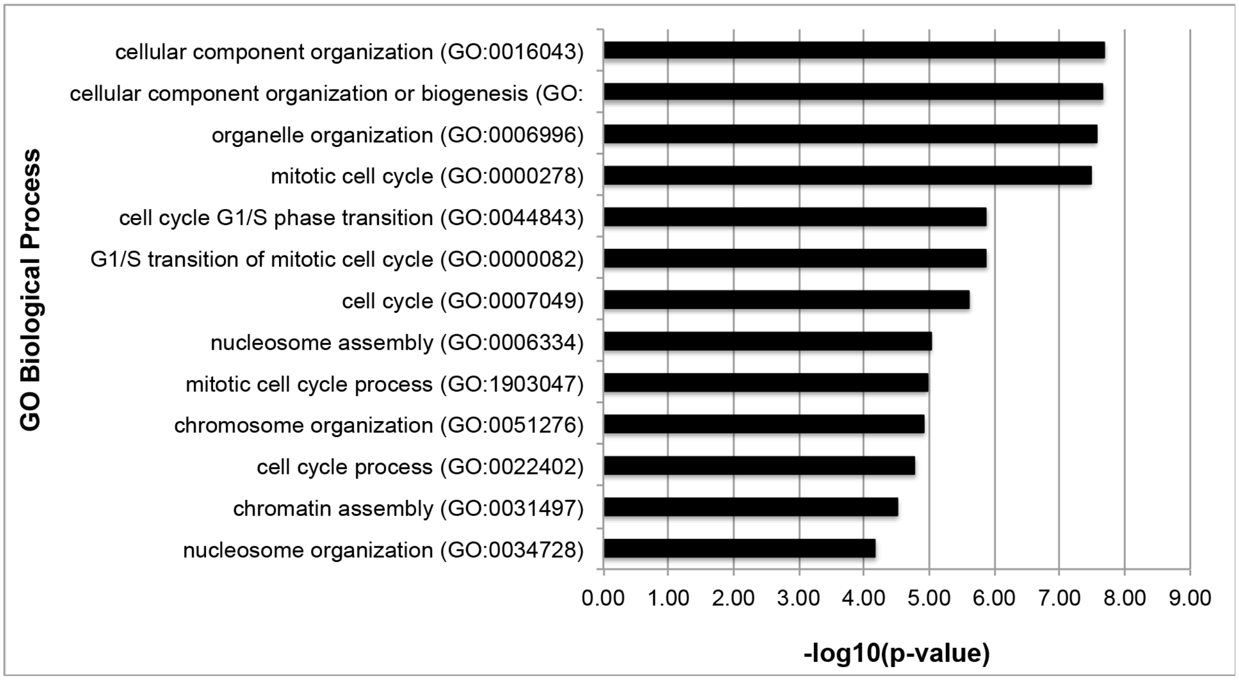

Supplementary Figure S5: GO Biological Processes enriched in overlapping transcripts differentially expressed in *TP53* mutant and *TP53* wild-type fibroblast cell lines transfected with miR-34A mimic. The –log10(*p*-value) of enrichment is given on this horizontal axis and GO biological processes is annotated on the vertical axis.

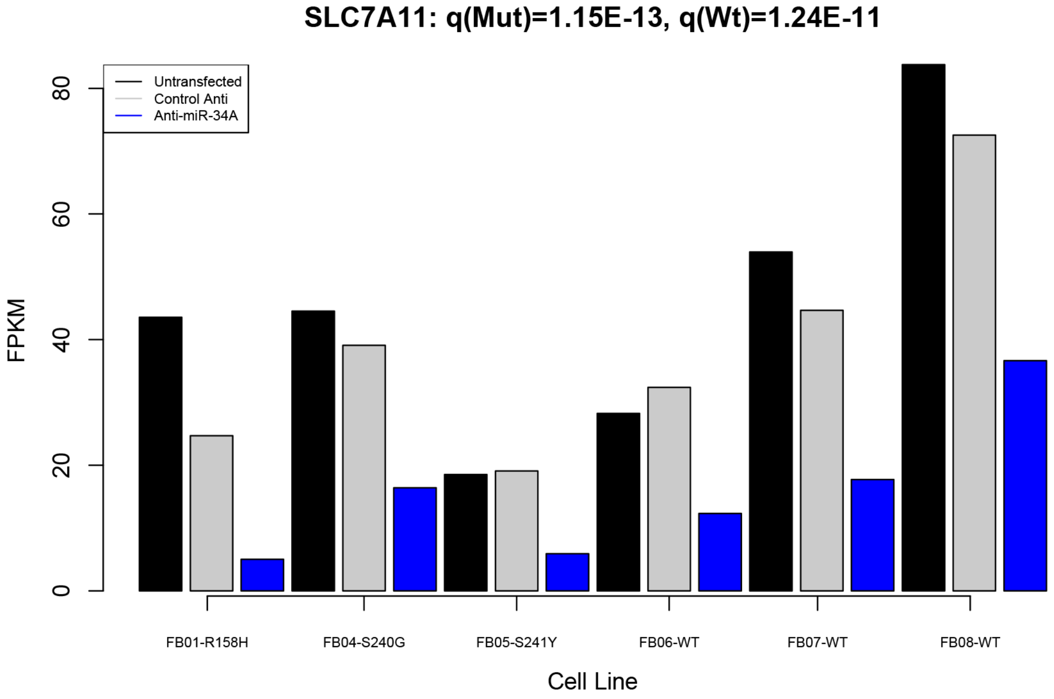

Supplementary Figure S6: Transcript expression of components of SLC7A11 in *TP53* mutant and wild-type cell lines transfected with anti-miR-34A. In each plot, the respective cell line per three bars is provided on the horizontal axis, below the cell line transfected with control anti-miR (grey). Black bars indicate untransfected lines and blue indicate cell lines transfected with anti-miR-34A. The vertical axis depicts the FPKM expression value for SLC7A11 across all cell lines at each transfection condition

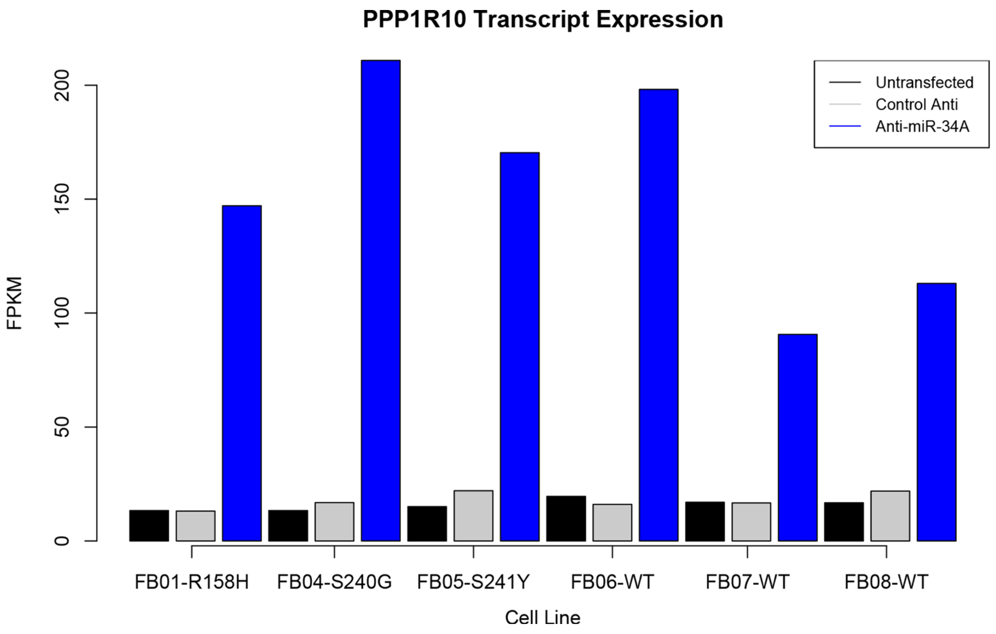

Supplementary Figure S7: Transcript expression of components of PPP1R10 in *TP53* mutant and wild-type cell lines transfected with anti-miR-34A. In each plot, the respective cell line per three bars is provided on the horizontal axis, below the cell line transfected with control anti-miR (grey). Black bars indicate untransfected lines and blue indicate cell lines transfected with anti-miR-34A. The vertical axis depicts the FPKM expression value for PPP1R10 across all cell lines at each transfection condition.

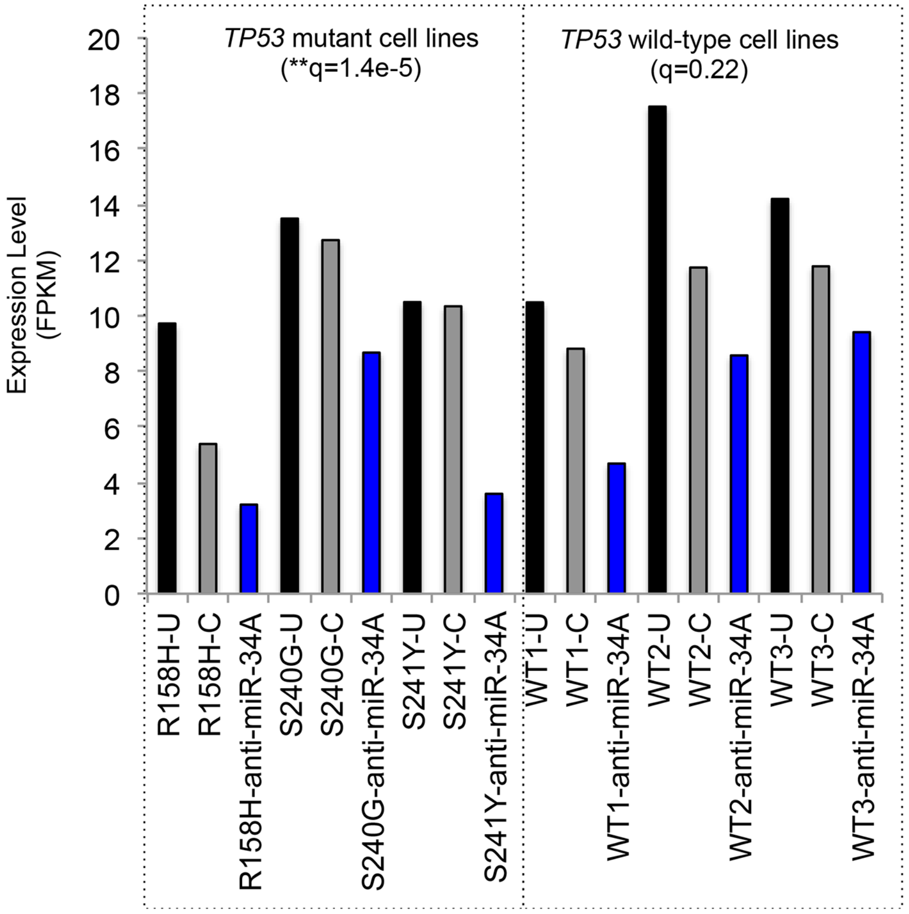

Supplementary Figure S8: TP53 transcript levels in *TP53* mutant and wild-type cell lines following tranfection with an anti-miR-34A. Black bars indicate untransfected lines and blue indicate cell lines transfected with anti-miR-34A. The vertical axis depicts the FPKM expression value for *TP53* across all cell lines at each transfection condition. (U = untransfected, C = control anti-miR; 'WT' in the cell line IDs on the horizontal axis indicate 'wild-type').

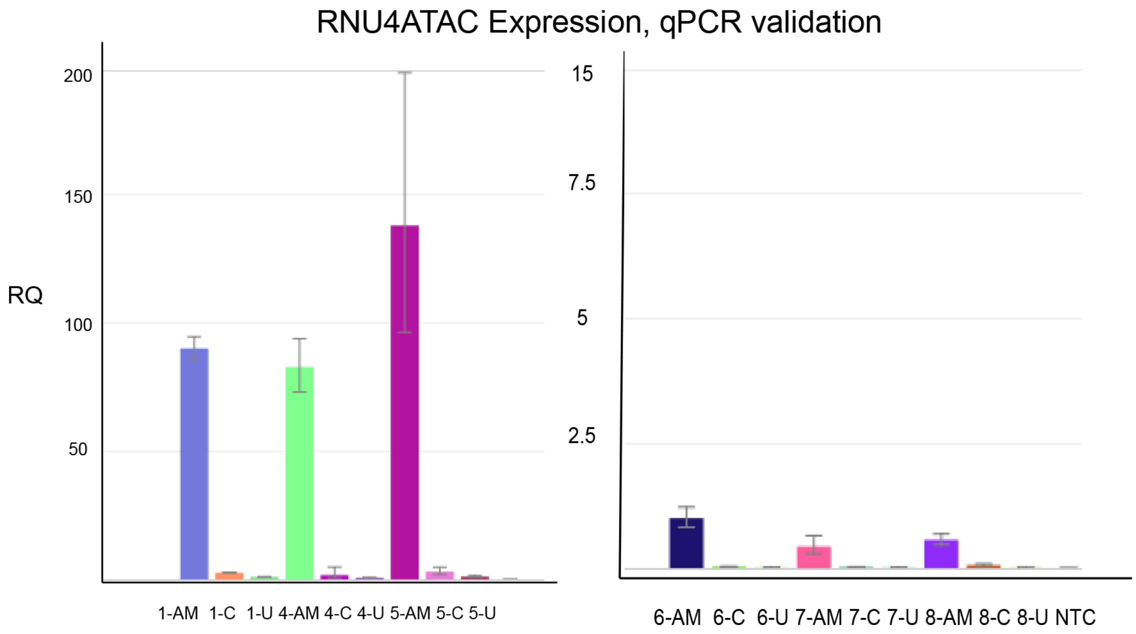

Supplementary Figure S9: qPCR validation of RNU4ATAC expression in *TP53* mutant and *TP53* wild-type cell lines. Expression levels were measured by qPCR; RQ = relative quantification; all transcripts were normalized to 18S Ct values. Error bars denote standard error of technical replicates (*n* = 3).

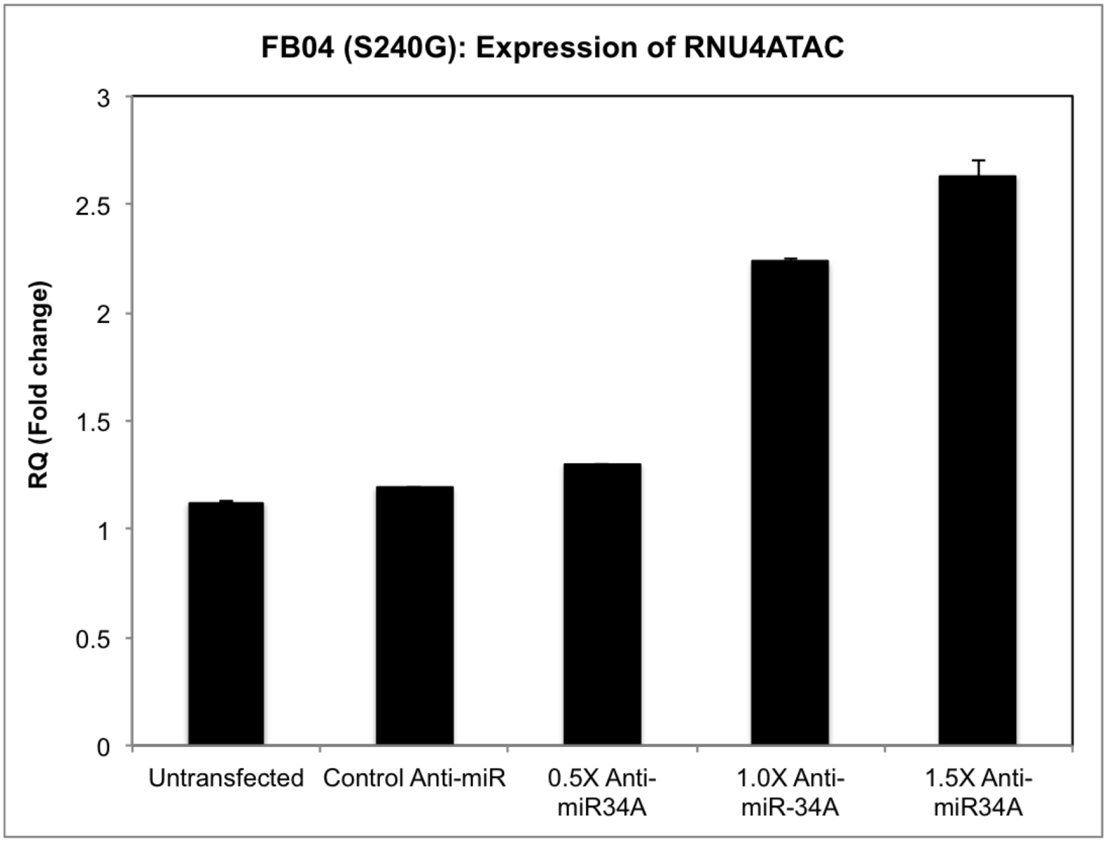

Supplementary Figure S10: RNU4ATAC expression by qPCR in FB04 (S240G) cell line using increasing doses of anti-miR-34A oligonucleotide. RQ = relative quantification; all transcripts were normalized to 18S Ct values. Error bars denote standard error of technical replicates (*n* = 3).

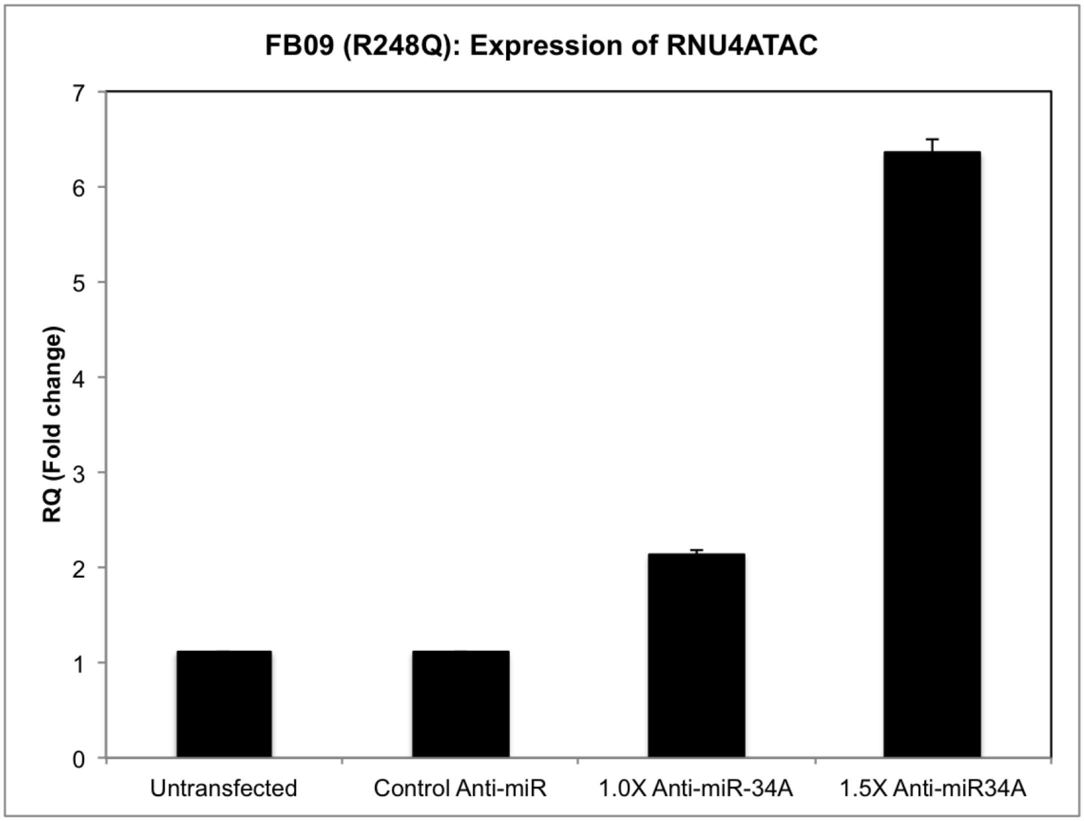

Supplementary Figure S11: RNU4ATAC expression by qPCR in FB09 (R248Q) cell line using increasing doses of anti-miR-34A oligonucleotide. RQ = relative quantification; all transcripts were normalized to 18S Ct values. Error bars denote standard error of technical replicates (*n* = 3).

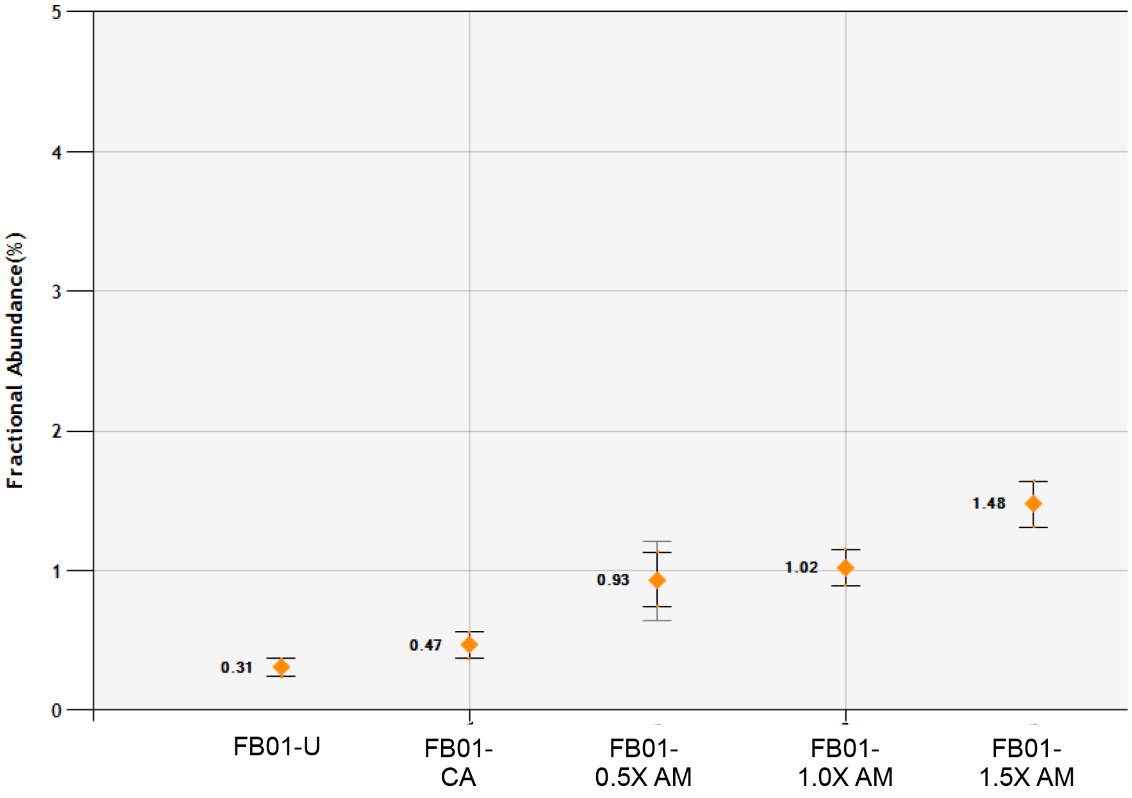

Supplementary Figure S12: LINC-PINT expression in FB01 cell line (R158H) by digital droplet PCR (ddPCR) in FB01 (R158H) cell line. Fractional transcript abundance per 1uL of input RNA is shown. Error bars denote standard deviation of technical replicates (*n* = 4).

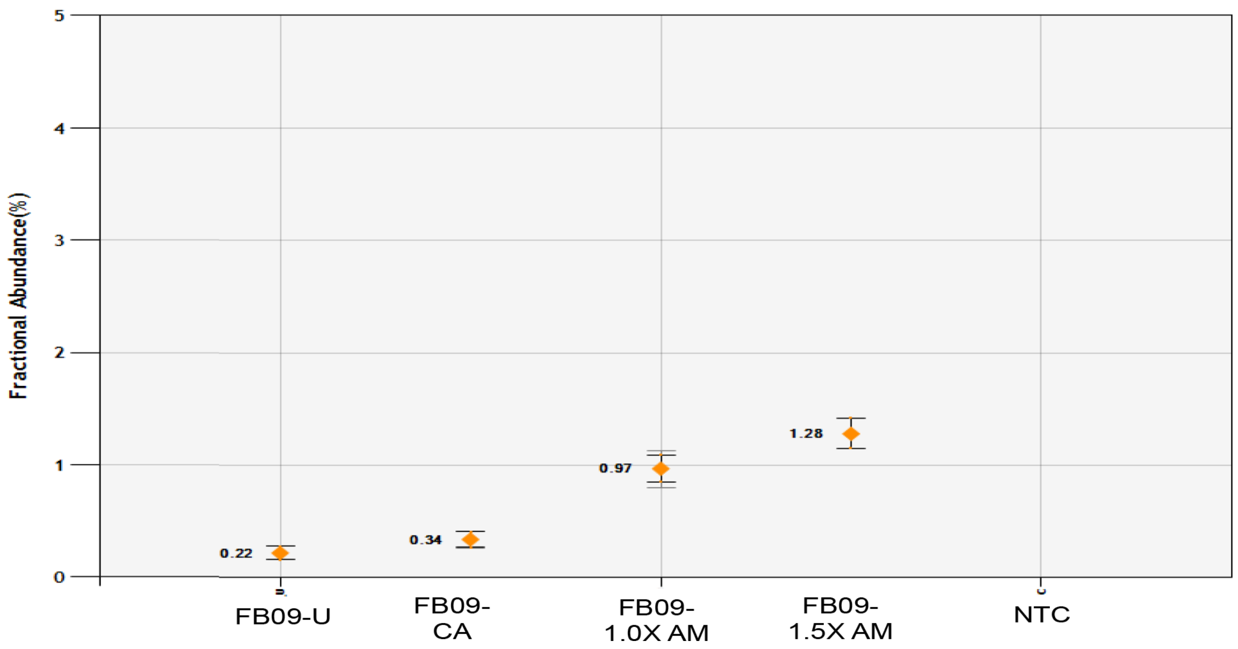

Supplementary Figure S13: LINC-PINT expression in FB09 cell line (R248Q) by digital droplet PCR (ddPCR) in FB01 (R158H) cell line. Fractional transcript abundance per 1uL of input RNA is shown. Error bars denote standard deviation of technical replicates ($n = 4$).

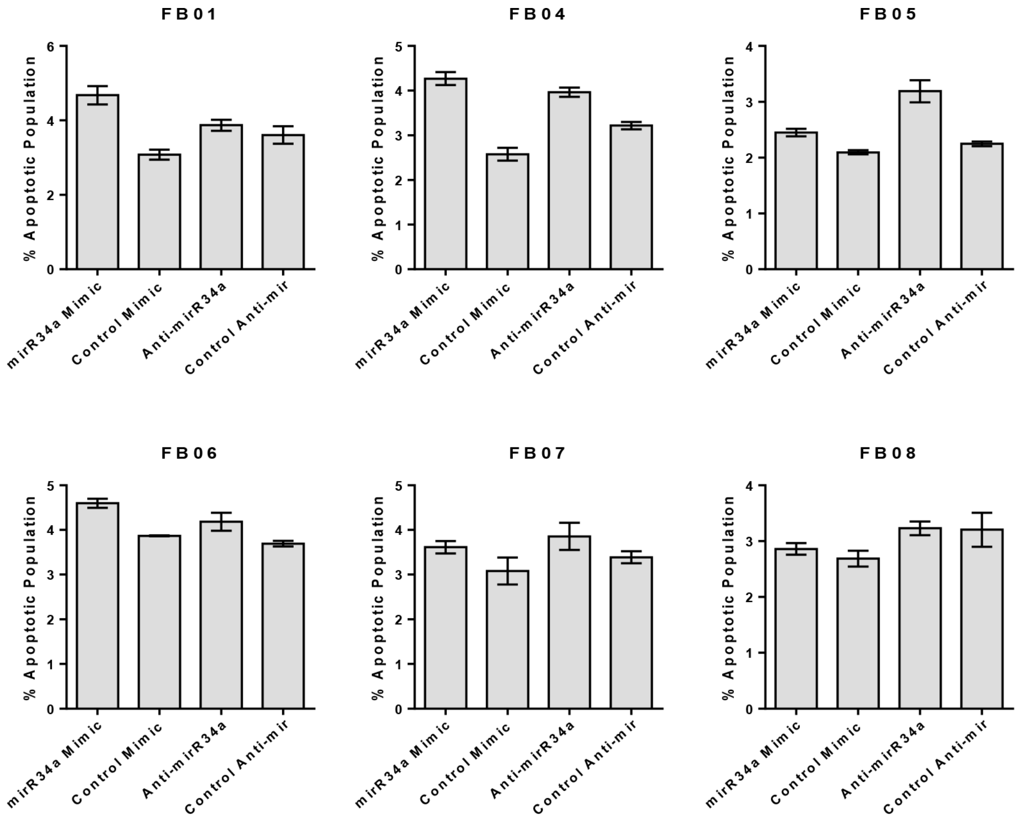

Supplementary Figure S14: Bar graphs of apoptotic cell fraction in LFS and *TP53* wild-type fibroblast cell lines transfected with miR-34A mimics or anti-miR-34A oligonucleotides. Vertical axis depicts the fraction of cells that were apoptotic (double positive Annexin-V/7AAD stain). The transfection agent used is identified on the horiztonal axis of each plot. FB01, FB04 and FB05 are primary fibroblast cell lines derived from LFS patients. FB07-08 are primary fibroblast cell lines derived with TP53 wild-type patients. Error bars denote standard deviation of technical replicates ($n = 3$).

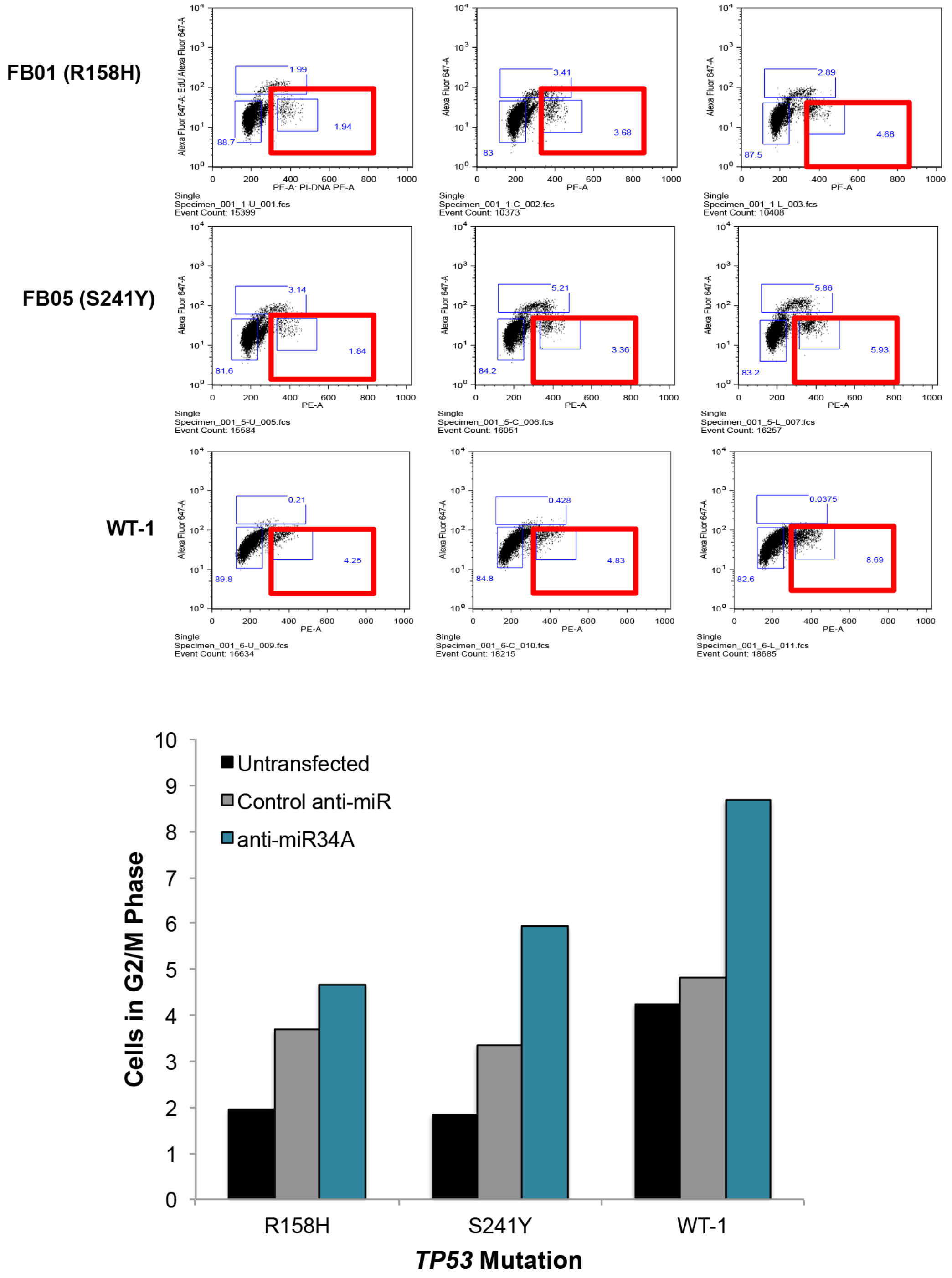

Supplementary Figure S15: (A–B) Experimental replicate of cell cycle analysis assays using three cell lines. Panel A shows the flow cytometry curves, with red boxes delineating proportion of cells in G2/M phase. Panel B depicts a bar graph of the fraction of cells in G2/M for each cell line and each experimental condition.

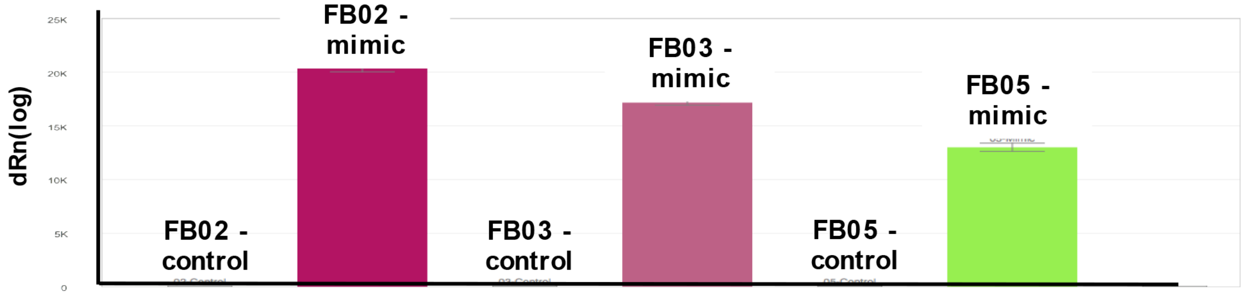

Supplementary Figure S16: qPCR validation of transfection efficiency in 3 cell lines.

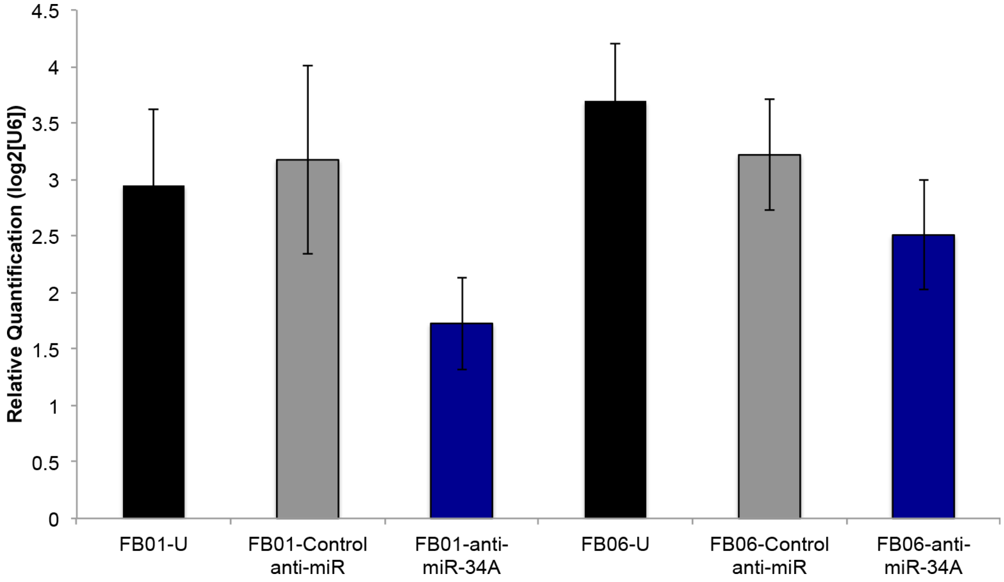

Supplementary Figure S17: qPCR validation of decreased miR-34A expression following transfection with an anti-miR-34A inhibitor (anti-miR). Expression levels of miR-34A are shown for each transfected condition in the FB01 cell line (R158H) and FB06 cell line (TP53 wild-type). Relative log2 miR-34A transcript expression is shown on the vertical axis, relative to snoU6 endogenous control (U = untransfected). Error bars denote standard deviation of technical replicates (n = 6). miR-34A levels are decreased relative to untransfected and control transfected cells for both cell lines (**$p < 0.001$ for all comparisons; FB01: U vs anti-miR: $p = 1.42e\text{-}5$; FB01 CA vs. anti-miR: $p = 9.5e\text{-}6$; FB06: U vs anti-miR: $p = 1.31e\text{-}7$; FB06: CA vs anti-miR: $p = 1.46e\text{-}10$).

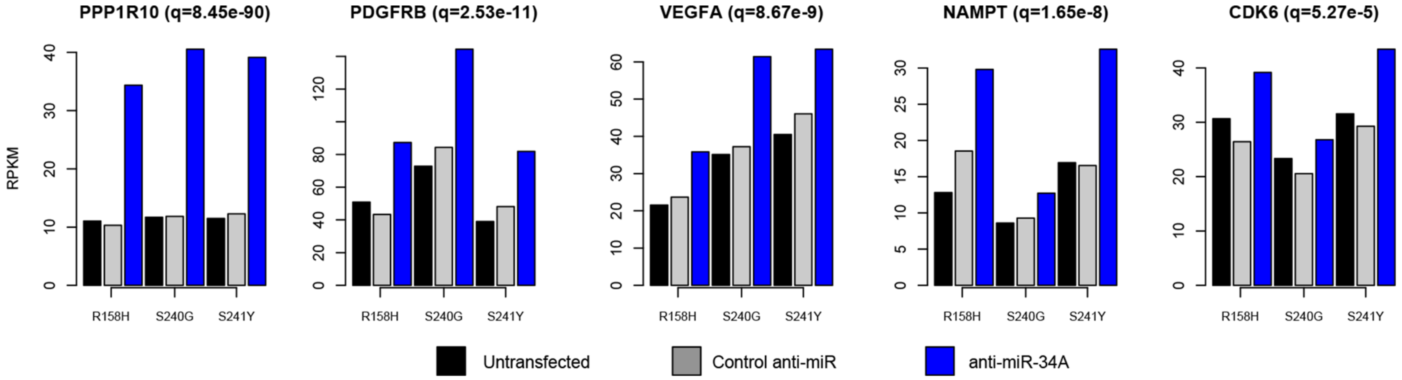

Supplementary Figure S18: Barplots depicting expression changes of known miR-34A target transcripts following transfection with hsa-miR-34A-5p inhibitor in 3 LFS cell lines (FB01, FB04, FB05). In each plot, the respective cell line per triplet bars (black, grey and blue bars) is provided on the horizontal axis, below the cell line transfected with control anti-miR (grey). Black bars indicate untransfected lines and blue indicate cell lines transfected with hsa-miR-34A-5p inhibitor. The vertical axis depicts the FPKM expression value for each transcript across all cell lines at each transfection condition.

Supplementary Table S1: Non-exhaustive list of cancers in which miR-34A deregulation is implicated

Malignancy	Reference
Acute myeloid leukemia	Rucker F. G. et al., 2013
Bladder carcinoma	Vinall R. L. et al., 2012
Brain cancer	Guessous F. et al., 2011
Brain glioma	Silber J. et al., 2012
Breast cancer	Kastl L. et al., 2012
Carcinoma	Pang R. T. et al., 2010
Cervical cancer	Wang X. et al., 2009
Choriocarcinoma	Pang R. T. et al., 2010
Chronic lymphocytic leukemia	Dijkstra M. K. et al., 2008
Colorectal cancer	Nugent M. et al., 2012
Esophageal cancer	Chen X. et al., 2012
Glioblastoma	Genovese G. et al., 2012
Head and neck cancer	Kumar B. et al., 2012
Hepatocellular carcinoma	Pineau P. et al., 2010
Laryngeal carcinoma	Shen Z. et al., 2012
Leukemia	Zauli G. et al., 2011
Lung cancer	Duan W. et al., 2010
Lung small cell carcinoma	Lee J. H. et al., 2011
Lymphoblastic leukemia	Mraz M. et al., 2009
Lymphoma	Craig V. J. et al., 2011
Medulloblastoma	De Antonellis P. et al., 2011
Melanoma	Heinemann A. et al., 2012
Myelodysplastic syndromes	Dostalova Merkerova M. et al., 2011
Neuroblastoma	Chakrabarti M. et al., 2012
Non-small cell lung carcinoma	Ji X. et al., 2012
Osteosarcoma	Yan K. et al., 2012
Ovarian cancer	Vogt M. et al., 2011
Ovarian papillary neoplasm	Taylor D. D. et al., 2008
Pancreatic cancer	Jamieson N. B. et al., 2012
Peripheral primitive neuroectodermal tumor	Nakatani F. et al., 2012
Prostate cancer	Kong D. et al., 2012
Renal cell carcinoma	Yamamura S. et al., 2012
Retinoblastoma	Jo D. H. et al., 2011
Sarcoma	Vogt M. et al., 2011
Squamous cell carcinoma	Chen X. et al., 2012
Squamous cell neoplasm	Scapoli L. et al., 2011
Stomach cancer	Ji Q. et al., 2008

Supplementary Table S2: Cells from which RNA was harvested for RNA-Sequencing

Cell Line	Transfection Condition	RNA-Seq ID
FB01	Untransfected	1A
	Control Mimic	1B
	Control Anti-miR	1C
	miR-34A Mimic	1D
	Anti-miR-34A	1E
FB04	Untransfected	4A
	Control Mimic	4B
	Control Anti-miR	4C
	miR-34A Mimic	4D
	Anti-miR-34A	4E
FB05	Untransfected	5A
	Control Mimic	5B
	Control Anti-miR	5C
	miR-34A Mimic	5D
	Anti-miR-34A	5E
FB06	Untransfected	6A
	Control Mimic	6B
	Control Anti-miR	6C
	miR-34A Mimic	6D
	Anti-miR-34A	6E
FB07	Untransfected	7A
	Control Mimic	7B
	Control Anti-miR	7C
	miR-34A Mimic	7D
	Anti-miR-34A	7E
FB08	Untransfected	A8
	Control Mimic	B8
	Control Anti-miR	C8
	miR-34A Mimic	D8
	Anti-miR-34A	E8

Supplementary Table S3: Number of differentially expressed RNA-genes and protein-coding transcripts by condition determined by EdgeR

TP53 Status	Condition	No. Iranscripts
	U_MT Vs. WT	136
Mutant	m_cm	657
Mutant	am_cam	2631
Mutant	Common-m & m	155
Mutant	m_cm_unique	502
Mutant	am_cam_unique	2576
Wild-Type	m_cm	196
Wild-Type	am_cam	357
Wild-Type	Common-m & m	3
Wild-Type	m_cm_unique	193
Wild-Type	am_cam_unique	354
	Common-MT and WT Mimic	128
Mutant	Mimic_Unique	589
Wild-Type	Mimic_Unique	68
	Common-MT and WT Anti	324
Mutant	Anti_Unique	2307
Wild-Type	Anti_Unique	33

(U = untransfected; MT = Mutant; WT = wild-type; m = mimic; cm = control mimic; am: anti-miR; cam = control anti-miR).

Supplementary Table S4: Number of differentially expressed RNA-genes and protein-coding transcripts by condition determined by Cufflinks

Cufflinks Summary FDR < 0.05

	U_MT vs. WT	421
Mutant	m_cm	101
Mutant	am_cam	592
Mutant	Common-m & am	26
Mutant	m_cm_unique	75
Mutant	am_cam_unique	566
Wild-Type	m_cm	0
Wild-Type	am_cam	71
Wild-Type	Common-m & am	0
Wild-Type	m_cm_unique	-
Wild-Type	am_cam_unique	71
	Common-MT and WT Mimic	-
Mutant	Mimic_Unique	101
Wild-Type	Mimic_Unique	-
	Common-MTO and WT Anti	50
Mutant	Anti_Unique	542
Wild-Type	Anti_Unique	21

(U = untransfected; MT = Mutant; WT = wild-type; m = mimic; cm = control mimic; am: anti-miR; cam = control anti-miR).

Supplementary Table S5: Histone H1 isoforms differentially expressed in *TP53* mutation fibroblast cell lines, relative to wild-type lines, at an FDR< 0.01

Gene	*p*-value	FDR	FB01-R158H	FB04-S240G	FB05-S241Y	FB06-WT	FB07-WT	FB08-WT
HIST1H1D	5.02E-05	0.00950858	76.7611583	47.0321032	58.6672818	69.640158	140.148255	37.9547678
HIST1H2AB	2.18E-05	0.0055459	84.5379309	75.4419374	83.2782844	28.6516101	48.7791892	10.9964423
HIST1H2AH	9.50E-06	0.00325831	264.156139	250.16283	321.104153	71.2595852	143.620196	49.0212283
HIST1H2AI	3.86E-05	0.00796446	220.548185	160.219299	212.992747	40.8198589	123.463965	30.8994436
HIST1H2AK	1.79E-06	0.00100528	86.2352537	55.0200687	80.7199983	28.1602958	38.1717903	13.8830461
HIST1H2AL	1.53E-05	0.004371	348.949511	246.377914	290.313314	77.1462466	203.901705	64.4194565
HIST1H2BF	2.86E-05	0.00641785	251.232645	202.284732	166.935645	36.1091533	65.6099053	49.4565156
HIST1H2BG	1.39E-05	0.00409764	380.211746	307.177467	357.975645	92.6754949	209.656059	40.3673732
HIST1H2BH	1.14E-05	0.00371916	205.267655	263.581632	330.880259	91.3491704	193.836439	72.0754665
HIST1H2BM	5.24E-06	0.0021451	228.969314	210.398105	228.832724	43.6639995	65.2382665	34.8769207
HIST1H3A	2.07E-05	0.00546266	128.011216	60.1023754	161.968779	29.1190724	78.9271345	23.0065594
HIST1H3C	4.23E-05	0.00838627	548.35872	401.440679	529.657537	118.169712	283.628917	79.343345
HIST1H3E	1.25E-07	9.94E-05	145.836625	105.355401	150.677104	19.2656208	65.6983832	30.7110025
HIST1H3F	1.41E-05	0.00409764	421.57233	313.412489	387.870827	79.64552	107.069354	57.8390171
HIST1H3H	1.95E-06	0.0010115	142.070745	209.935536	280.580726	54.0337868	129.265546	34.9011072
HIST1H3J	2.46E-05	0.00583851	160.289343	136.32322	92.6015296	35.2165179	91.3649666	24.064575
HIST1H4A	1.19E-07	9.77E-05	88.4011295	73.8194207	87.2571116	21.2986928	32.6094165	10.5023064
HIST1H4B	3.17E-05	0.00699157	196.564379	149.945654	244.35628	88.9449443	112.052231	37.9009927
HIST1H4L	2.10E-05	0.00549228	206.266965	219.451012	152.392934	57.5185038	123.659304	59.4262095

FPKM (Fragments Per Kilobase of transcript per Million mapped reads) values are listed for each cell cell for the respective gene.

Supplementary Table S6: Transcripts that are highly differentially expressed in both TP53 mutant and TP53 wild-type cells transfected with miR-34A mimic

AKT3	CTDSP1	HIST1H2AJ	LZTFL1	PGM2L1	SLC27A4	UHRF1
ANGPT1	DDX18	HIST1H2AL	MAP2K1	PHF19	SLC29A1	VAMP4
ARL1	DRAM2	HIST1H2BJ	MAP2K4	PKMYT1	SLC2A3	WSB1
ARRDC4	E2F1	HIST1H2BK	MCFD2	PLAG1	SMAP1	WWTR1
ASF1B	E2F8	HIST1H2BL	MCM2	POLE	SSFA2	ZBTB18
ATP11B	ERLIN1	HIST1H2BM	MCM3	PTPMT1	STAG2	ZBTB47
AXL	ESPL1	HIST1H3B	MCM5	PTPN13	STX12	ZDHHC18
BOD1	ESYT2	HIST1H3C	MCM7	PVRL1	TAPT1	ZHX1
BRWD3	FAM167A	HIST1H3D	MED21	RBPJ	TBC1D13	
C1D	FANCA	HIST1H3F	MET	RFK	TBL1XR1	
C21orf58	FOSL1	HIST1H3G	MSRB3	RNF6	TCF19	
C6orf120	G3BP2	HIST1H3H	MYBL2	RRAS	TMEM173	
C7orf49	GABPA	HIST1H4D	NGRN	RRM2	TMEM184B	
CALU	GMNN	HIST1H4E	NKIRAS1	RSL24D1	TMEM241	
CDC25A	GOPC	HIST1H4L	NRAS	SAMD8	TMEM65	
CDK4	GSG2	HIST2H2BF	NRBF2	SBNO1	TMEM67	
CDT1	HDAC1	KIF18B	NUMBL	SEL1L	TOMM20	
CENPO	HIST1H1B	KIFC1	NUP62	SEMA3C	TONSL	
CFL2	HIST1H1E	LETM1	ORC1	SH3GL1	TP53INP1	
CNOT7	HIST1H2AI	LYSMD3	PDCD6	SLC25A22	TTC30A	

Supplementary Table S7: Suppressed transcripts containing the miR-34A complimentary seed sequence in *TP53* mutant cell lines treated with hsa-miR-34A-5p mimic

gene	ACTGCC?	logFC	FDR
KIF26A	YES	–5.6031008	0.00019818
THBD	YES	–1.5929771	1.55E-10
HPDL	YES	–1.4841305	0.00068574
FAM167A	YES	–1.2952531	2.92E-20
GMNN	YES	–1.2744642	3.42E-15
TMED8	YES	–1.2460065	2.12E-09
HIST1H3J	YES	–1.2347617	1.18E-10
HIST1H4A	YES	–1.2338562	1.02E-14
HIST1H1A	YES	–1.2131304	4.07E-06
SGPP1	YES	–1.2078861	2.42E-26
CCNE2	YES	–1.2075501	1.37E-06
HIST1H4I	NO	–1.2075498	3.40E-09
HIST1H2BL	NO	–1.1705875	4.97E-09
HIST1H2BE	YES	–1.1497083	3.42E-09
CDC25A	YES	–1.1465331	1.32E-09
HIST1H3H	NO	–1.1456572	5.97E-12
HIST2H3D	YES	–1.135703	8.10E-07
HIST1H2AB	NO	–1.134229	8.76E-12
FAM111B	NO	–1.1299169	8.45E-07
HIST1H3F	NO	–1.1183726	8.88E-10

Supplementary Table S8: Suppressed transcripts containing the miR-34A complimentary seed sequence in *TP53* wild-type cell lines treated with hsa-miR-34A-5p mimic

gene	ACTGCC	logFC	FDR
SLC29A1	YES	–1.1884835	5.87E-05
HIST1H2BL	NO	–1.0930792	7.39E-05
TONSL	YES	–1.0092249	0.00090603
ASF1B	NO	–1.0036141	0.0006666
CDIP1	YES	–0.9596118	0.00015588
C21orf58	NO	–0.9464261	0.00025188
HIST1H3F	NO	–0.9358122	0.00011235
HIST1H3B	NO	–0.9294908	0.00045
FAM167A	YES	–0.9287398	8.79E-05
HIST2H2BF	NO	–0.8874128	0.00015778
SLC25A22	YES	–0.8567657	0.00038556
E2F1	NO	–0.8487724	0.0005459
HIST1H2AI	NO	–0.82817	0.00014944
MCM3	NO	–0.813352	2.83E-05
HIST1H1B	NO	–0.768363	0.00034949
AXL	YES	–0.7366334	0.00011235
CDK4	NO	–0.6867536	9.37E-05

Supplementary Table S9: Characterized or predicted lincRNAs regulated by miR-34A in *TP53* mutant cell lines

gene	EGID	am_cam_LR	am_cam_*P*Value	am_cam_FD
LINC-PINT	378805	574.5745021	5.68E-127	2.88E-123
LOC644656	644656	474.0475606	4.22E-105	1.78E-101
LINC00936	338758	371.0286995	1.12E-82	2.83E-79
MIR100HG	399959	370.6336456	1.36E-82	3.14E-79
NS3BP	171391	332.1279395	3.31E-74	5.99E-71
MIAT	440823	330.0461248	9.39E-74	1.59E-70
LOC284454	284454	268.1491525	2.87E-60	3.84E-57
LOC400684	400684	263.7290353	2.64E-59	3.35E-56
LOC100272217	100272217	261.2440532	9.19E-59	1.11E-55
FRMD6-AS1	145438	260.5888713	1.28E-58	1.47E-55
LINC01126	100129726	253.3655456	4.79E-57	4.87E-54
STAM-AS1	102723166	248.018317	7.02E-56	6.85E-53
LOC101927415	101927415	225.4079315	5.98E-51	5.23E-48
LINC00441	100862704	225.1030522	6.97E-51	5.90E-48
KMT2E-AS1	100216545	220.0493248	8.82E-50	6.99E-47
LOC101928736	101928736	216.9251626	4.24E-49	3.26E-46
C12orf61	NA	215.9358295	6.96E-49	5.20E-46
LOC100129917	100129917	215.2926096	9.62E-49	6.97E-46
LOC101928068	101928068	213.9745001	1.87E-48	1.31E-45
MAN1B1-AS1	100289341	213.3515087	2.55E-48	1.70E-45
LOC101927814	101927814	211.4063793	6.78E-48	4.41E-45
UBAC2-AS1	100289373	204.9958059	1.70E-46	1.08E-43
LOC101927780	101927780	199.303543	2.96E-45	1.79E-42
CAHM	100526820	198.5372863	4.36E-45	2.57E-42
CPEB2-AS1	441009	197.8035866	6.30E-45	3.63E-42
HCG25	414765	196.1184259	1.47E-44	8.28E-42
FLJ38576	651430	195.3140965	2.20E-44	1.21E-41
LOC54944	NA	189.9619882	3.24E-43	1.68E-40
BRWD1-IT2	103091865	188.2538811	7.65E-43	3.88E-40

Supplementary Table S10: Characterized or predicted lincRNAs regulated by miR-34A in *TP53* wild-type cell lines

gene	EGID	am_cam_LR	am_cam_*P*Value	am_cam_FDR
LINC-PINT	378805	381.8643292	4.89E-85	1.24E-80
LOC644656	644656	119.7353594	7.23E-28	6.11E-24
LOC344887	344887	69.94253035	6.11E-17	2.21E-13
LINC00936	338758	58.27640275	2.28E-14	3.21E-11
SPACA6P-AS	102238594	56.07307338	6.98E-14	7.70E-11
CPEB2-AS1	441009	55.53061977	9.20E-14	9.34E-11
LOC400684	400684	55.53371428	9.19E-14	9.34E-11
LOC101927814	101927814	51.32297876	7.84E-13	6.85E-10
STAM-AS1	102723166	48.45045765	3.39E-12	2.63E-09
HEXA-AS1	80072	48.2415082	3.77E-12	2.81E-09
MIAT	440823	48.16623803	3.92E-12	2.84E-09

NEAT1	283131	47.44178139	5.67E-12	3.99E-09
C12orf61	NA	47.14750687	6.58E-12	4.51E-09
BCYRN1	618	46.40988005	9.59E-12	6.40E-09
FRMD6-AS1	145438	46.3259134	1.00E-11	6.51E-09
MGC45800	90768	45.09756814	1.87E-11	1.16E-08
LOC101927415	101927415	44.73577041	2.25E-11	1.36E-08
LINC00472	79940	44.54903341	2.48E-11	1.46E-08
LOC284454	284454	44.45702603	2.60E-11	1.50E-08
LOC100630918	100630918	42.49563696	7.08E-11	3.99E-08
CTB-113P19.1	101927096	41.86646707	9.77E-11	5.15E-08
LOC100129917	100129917	41.83264304	9.94E-11	5.15E-08

Supplementary Table S11: De-repression of known miR-34A target transcripts following transfection with hsa-miR-34A-5p inhibitor in 3 LFS cell lines (FB01, FB04, FB05)

Gene ID	Log (fold change)	FDR (q)
PPP1R10	1.727372614	8.45E-90
PDGFRB	0.848608276	2.53E-11
VEGFA	0.59431805	8.67E-09
NAMPT	0.712469046	1.65E-08
CDK6	0.508272026	5.27E-05
MET	0.500283172	0.000296188
GAS1	0.647907363	0.00067168
AXL	0.449788043	0.000685509
CCND1	0.474472527	0.00079473
YY1	0.295662589	0.013352936
CSF1R	0.828064395	0.035190892
MYC	0.252651332	0.037723845

Supplementary Data: Differential Expression Analysis. See Supplementary Data
